# 9-*cis*-Epoxycarotenoid Dioxygenase 3 Regulates Plant Growth and Enhances Multi-Abiotic Stress Tolerance in Rice

**DOI:** 10.3389/fpls.2018.00162

**Published:** 2018-03-06

**Authors:** Yuan Huang, Yiming Guo, Yuting Liu, Feng Zhang, Zhikui Wang, Hongyan Wang, Feng Wang, Dongping Li, Dandan Mao, Sheng Luan, Manzhong Liang, Liangbi Chen

**Affiliations:** Hunan Province Key Laboratory of Crop Sterile Germplasm Resource Innovation and Application, College of Life Science, Hunan Normal University, Changsha, China

**Keywords:** *OsNCED3*, abscisic acid (ABA), CRISPR/Cas9 system, seed germination, growth, abiotic stress, leaf senescence

## Abstract

Although abscisic acid (ABA) is an important hormone that regulates seed dormancy, stomatal closure, plant development, as well as responses to environmental stimuli, the physiological mechanisms of ABA response to multiple stress in rice remain poorly understood. In the ABA biosynthetic pathway, 9-*cis*-epoxycarotenoid dioxygenase (NCED) is the key rate-limiting enzyme. Here, we report important functions of *OsNCED3* in multi-abiotic stress tolerance in rice. The *OsNCED3* is constitutively expressed in various tissues under normal condition, Its expression is highly induced by NaCl, PEG, and H_2_O_2_ stress, suggesting the roles for *OsNCED3* in response to the multi-abiotic stress tolerance in rice. Compared with wild-type plants, *nced3* mutants had earlier seed germination, longer post-germination seedling growth, increased sensitivity to water stress and H_2_O_2_ stress and increased stomata aperture under water stress and delayed leaf senescence. Further analysis found that *nced3* mutants contained lower ABA content compared with wild-type plants, overexpression of *OsNCED3* in transgenic plants could enhance water stress tolerance, promote leaf senescence and increase ABA content. We conclude that *OsNCED3* mediates seed dormancy, plant growth, abiotic stress tolerance, and leaf senescence by regulating ABA biosynthesis in rice; and may provide a new strategy for improving the quality of crop.

## Introduction

Abscisic acid (ABA) is a sesquiterpenoid that plays a critical role in seed dormancy, stomatal closure, plant development, and biotic/abiotic stress tolerance during the plant life cycle (Nambara and Marion-Poll, 2005). Furthermore, the ABA levels in plant tissues are usually dynamic in order to respond to physiological changes and environmental stimuli. For instance, in relation to shoot and root growth, ABA is generally considered to be an inhibitor in well-watered condition, but normal ABA levels are necessary to maintain root growth (in *vp5* or *vp14* mutants) under water stress, and normal ABA levels are necessary for shoot development, particularly leaf expansion (in *flc* or *aba2* mutants) under well-watered conditions (Sharp et al., 2000; Sharp and LeNoble, 2002; LeNoble et al., 2004). Under water stress, ABA is dramatically increased, and it regulates stomata closure in plants to reduce water loss. This is an ABA-dependent mechanism, which involves activating H_2_O_2_ production, which subsequently increases calcium levels in guard cells, which triggers stomatal pores closure (Tardieu and Davies, 1992; Wang and Song, 2008; Yao et al., 2013). Although the physiological importance of ABA in plant growth and abiotic stress tolerance has been well-recognized, the molecular mechanisms of ABA response to multiple stress in rice remain poorly understood.

The endogenous concentration of ABA in plant tissues is regulated by ABA biosynthesis (Ng et al., 2014). ABA is produced *de novo* in the ABA biosynthesis pathway, which originates from the catalysis of carotenoid precursors for several enzymes found in higher plants (Xu et al., 2013). To date, most ABA biosynthesis genes have been discovered and cloned, including those for zeaxanthin epoxidase (*ZEP*), 9-*cis*-epoxycarotenoid dioxygenase (*NCED*), and abscisic aldehyde oxidase (*AAO*) (Taylor et al., 2000; Hauser et al., 2011). The first committed step of ABA biosynthesis is the cleavage of 9-cis-violaxanthin or 9-cis-neoxanthin by NCED to produce xanthoxin (C_15_) (Tan et al., 1997; Milborrow, 2001). The first *NCED* gene to be identified and cloned was *VP14* in maize (Schwartz et al., 1997), and subsequently, *NCED* genes were isolated from other plant species (Priya and Siva, 2015) such as tomato (Burbidge et al., 1999), avocado (Chernys and Zeevaart, 2000), *Arabidopsis* (Rock and Zeevaart, 1991; Tan et al., 2003), *Malus* (Xia et al., 2014), and *Brassica napus* (Xu and Cai, 2017).

Previous studies have shown that increased *NCED* transcript levels could promote ABA biosynthesis and increase ABA accumulation in plants (Qin and Zeevaart, 2002; Martinez-Andujar et al., 2011). Several *NCED* mutants have been identified and studied; many have reduced resistance to severe environmental conditions or abnormal and defective morphology. The *VP14* from maize is expressed in embryos and roots and is strongly induced in leaves by water stress. *VP14* from maize is responsible for promoting seed dormancy and water stress resistance by controlling ABA levels in plants (Tan et al., 1997; Sharp and LeNoble, 2002). In addition, *BnNCED3* was isolated from *Brassica napus*, and ectopically expressed in Arabidopsis. The authors found that overexpression of *BnNCED3* elevated ABA levels in plants, delayed seed germination, reduced lateral root initiations, and promoted leaf senescence and an early flowering time (Xu and Cai, 2017). Furthermore, *NCED* is a multigene family; there are five *NCED* members confirmed in Arabidopsis, each of which is located in specific tissues, where they control ABA biosynthesis and regulate development (Tan et al., 2003). However, many of these proteins share redundant functions (Finkelstein, 2013). *AtNCED6* is constitutively expressed in the endosperm (Lefebvre et al., 2006; Martinez-Andujar et al., 2011), but *AtNCED9* is expressed both in the embryo and endosperm during seed development (Toh et al., 2007; Seo et al., 2016). *AtNCED5* is also expressed in the seed at later periods of development. *AtNCED5, AtNCED6*, and *AtNCED9* co-regulate seed development and dormancy (Frey et al., 2012). *AtNCED3* is predominantly induced by water stress and controls endogenous ABA content under water stress conditions (Endo et al., 2008; Hao et al., 2009), and thus, the *AtNCED3* T-DNA insertion mutant has a water deficiency-sensitive phenotype (Iuchi et al., 2001). *AtNCED5* and *AtNCED3* participate together in the water stress response in plants; moreover, *nced3* and *nced5* mutants suppress vegetative growth of Arabidopsis (Frey et al., 2012).

To date, five *NCED* genes have been found and implicated in ABA biosynthesis in rice (Zhu et al., 2009). Gene expression analysis showed that *OsNCED1* has the highest expression level in rice leaves, and acts as the housekeeping gene under normal conditions, but it is significantly suppressed by water stress (Ye et al., 2011). The expression levels of *OsNCED2* and *OsNCED3* are both related to delayed seed germination (Zhu et al., 2009; Song et al., 2012). Additionally, *OsNCED3, OsNCED4*, and *OsNCED5* are induced by water deficiency stress (Teng et al., 2014; Zhang et al., 2015). Although *OsNCED3* was ectopically expressed in Arabidopsis, and contributes to increased ABA accumulation, enhanced drought tolerance, and altered leaf morphology (Hwang et al., 2010), the native function of *OsNCED3* in rice is still unclear. In this study, we investigated the physiological roles of *OsNCED3* using the knock-out *nced3* mutants and *OsNCED3*-overexpressing transgenic rice plants. Our results suggest that *OsNCED3* regulates seed dormancy, stomata aperture, plant growth, abiotic stress tolerance, and leaf senescence by altering ABA accumulation in rice.

## Materials and methods

### Mutant generation methods

CRISPR/Cas9 system (provided by Professor Lijia Qu) was applied to generate *nced3* mutants. The *OsNCED3* coding sequence was selected to guide RNA design. According to NCBI blast (https://blast.ncbi.nlm.nih.gov/Blast.cgi) and CRISPR GE tool (http://skl.scau.edu.cn/) analysis, the specific 20 bp spacer sequences (GCCGCCCGCGCGCGCGCTGC) immediately before a PAM sequence(5′-NGG-3′) were selected and synthesized on the base of protocol (provided by Professor Lijia Qu) with additional *Bsa*I restriction site sequences at the pairs of sequences ends. The double-stranded spacers were generated by annealing and inserted into the *Bsa*I-digested pOS-sgRNA vector. Then this constructed pOS-sgRNA was implemented with the destination vector pH-Ubi-Cas9 through gateway cloning LR reaction (Invitrogen, Shanghai, USA). The resulting plasmids were sequenced.

The completed pH-Ubi-Cas9 constructs were then transformed into rice (*O. sativa L. japonica*) by *Agrobacterium*-mediated. The transgenic seedlings were grown in a chamber at 28°C under a 14 h light/10 h dark cycle or in a greenhouse with natural sunlight. For mutation detection, genomic DNA was extracted from whole mutant seedlings by using PlantGen DNA kit (CWbiotech, Beijing, China). Genomic DNA was amplified by using mutation detection primers which designed to flank the designated target site. Then, the sequence chromatograms were analyzed by DSDecode tool (http://dsdecode.scgene.com/) to check the genotype of the transgenic plants. The 35 T_0_ hygromycin-resistant transgenic plants have three genotypes including homozygous, bi-allelic and heterozygous lines. The two independent homozygous mutant lines from the T_1_ generation which were named *nced3-1* and *nced3-2* used for future study. The primers used for CRISPR/Cas9 (U3-NCED3-F/R) and mutation detection (NCED3-J-F/R) are listed in Table S3.

The transgenic plants off-target effects analysis was performed on the base of CRISPR GE tool (http://skl.scau.edu.cn/). The potential off-target sites were detected by using site-specific genomic PCR and sequenced to determine whether the potential off-target sites were also edited. The site-specific primers are listed in Table S3.

### Plant growth condition and treatment

All of the experiments were performed using *O. sativa* L. *japonica* seeds from the same harvest and storage conditions. Seeds were surface-sterilized in 75% (v/v) ethanol for 2 min and rinsed twice in sterile double-distilled water, and then in 2.5% (v/v) NaClO for 30 min. The seeds were then rinsed five times in sterile double-distilled water. The sterilized seeds were soaked in water at 30°C for 2 d and then germinated for 3 d at 28°C. Seedlings were grown in Hoagland's culture solution as described previously (Li and Cheng, 2015). Plants were cultured in a growth chamber at 28°C under a 14 h light/10 h dark photoperiod. For abiotic stress, three-leaf stage seedlings were placed into solutions with final concentrations of 25% PEG6000 (−0.51 Mpa, the solution was aerated in the experimental system), 150 mM NaCl and 100 mM H_2_O_2_. All treatments were repeated more than three times. The seedlings were harvested for RNA isolation or phenotype analyses.

For drought treatments at the seedling stage, the germinated seedlings were transplanted into a soil mix (2:1 soil:vermiculite) medium in oblong white resin pots (L33 × W19 × H14 cm). The soil was river alluvial paddy soil collected from experimental field of the Hunan Normal University, Changsha, 28°11′49″N, 112°58′42″E, Hunan province, China. The seedlings were grown to the three-leaf stage and then were subjected to withholding water for 15 d and photographed, followed watering was resumed for 7 d. The plants were photographed again and the number of surviving plants was scored. During the reproductive-stage water stress, the seedlings were grown in potted bucket (40 cm diameter, 40 cm height) until the inflorescence meristem appeared; then we withheld watering for 7 d until the leaves rolled. We photographed the plants at this time and then resumed watering to the mature stage and statistically assessed the seed set rate.

### Seed germination analysis

The harvested seeds from *nced3* mutants and WT rice plants were surface-sterilized and directly sown onto the sterile filter papers for a seed germination assay. Seeds were placed in a Polymethyl methacrylate germinating box and cultured in growth chamber (ZHUJIANG, Guangzhou, China) keep at 30°C. The germination ratio was based on radicles over 1 mm and was recorded after 2 d of imbibition. Moreover, we determined the shoot and root length. Three independent experiments were performed for this analysis.

### Quantitative real-time PCR analysis

Total RNA was extracted from rice seedlings or specific tissues at reproductive stage using Trizol according to the manufacturer's instructions (Invitrogen, Shanghai, USA). Approximately 1 μg of total RNA was used for first-strand cDNA synthesis with the Synthesis Kit (Thermo Fisher Scientific, USA). The reaction products were diluted ten-fold and used as the template for real-time PCR with three biological replicates using the SYBR Premix Ex Taq kit (Takara, Dalian, China). qRT-PCR was performed with an Applied Biosystems Quant Studio 5 (Thermo Fisher Scientific, USA) real-time PCR System. The gene-specific primers used in the qRT-PCR analysis are listed in Table S3.

### Generation of *OsNCED3* overexpression lines

The coding sequence of *OsNCED3* was amplified by the pHB-NCED3-F/R primers (Table S3) and then inserted into pHB binary vector behind 2 × 35S promoter. The recombinant construct pHB-35S::*OsNCED3* was transformed into calli of *O. sativa L. japonica* through *Agrobacterium* mediated transformation. The T_2_ generation transgenic seeds were screened through planted on MS medium containing 50 μg/L hygromycin (Hgr) for 7 d. The 100% resistance to Hgr were selected as homozygous lines.

### Gus staining and subcellular localization assay

For histochemical analysis, the *OsNCED3* promoter, a 2.27 kb genomic DNA fragment upstream region from the transcriptional initiation site was amplified by PCR using the primers 1301-OsNCED3-F/R (Table S3) and fused into pCambia1301 vector to drive the reporter gene β-glucuronidase (GUS). The Pro_*NCED*3_-GUS construct was transformed into rice (*O. sativa L. japonica*). The Pro_*NCED*3_-GUS transgenic plants (T_1_) were used to histochemical staining analysis. Histochemical staining was conducted as previously described (Li et al., 2015). The tissues of Pro_*NCED*3_-GUS transgenic plants (T_1_) were incubated in the GUS staining solution (50 mM sodium phosphate at pH 7.0, 10 mM EDTA, 0.5 mM potassium ferricyanide, 0.5 mM potassium ferrocyanide, 10% methanol, 0.1% Triton X-100, and 1 mg mL^−1^ of X-Gluc (Sangon, Shanghai, China) dissolved in N,N-dimethyl formamide) at 37°C for 10 h, the samples were incubated in 75% (v/v) ethanol to clear the chlorophyll. The chlorophyll-free samples were observed and imaged with OLYMPUS SZX7 and BX51 microscope.

To investigate the subcellular location of OsNCED3, the coding sequence for *OsNCED3* was amplified and ligated into the pEZS-eGFP vector. The recombinant vector pEZS-OsNCED3-eGFP was transformed into Arabidopsis protoplasts according to a protocol described previously (Meng et al., 2014). The GFP fluorescence in the transformed protoplasts was visualized with a confocal fluorescence microscope (Nikon A1, Japan).

### Analyses of proline content, electrolyte leakage, and water loss rate

The proline content determined as previously described (Bates et al., 1973). Approximately 0.5 g leaf segment samples from transgenic and WT plants were homogenized in 6 mL of 3% aqueous sulfosalicylic acid and centrifuged at 4,000 × g for 10 min. Then, 2 mL of supernatant was incubated with 2 mL of acid ninhydrin and 2 mL of glacial acetic acid in boiling water for 40 min, and cooled on ice to stop the reaction. For every reaction, 4 mL toluene was added and the reaction solution was oscillated for 30 min at room temperature. The absorbance was measured at 520 nm with a spectrophotometer.

The relative electrolyte leakage measurements were collected as previously described (Lou et al., 2017) with slight modification. The leaves were placed in tubes containing 20 mL of deionized water and then placed under a vacuum for 1 h. Samples were then sealed and placed at 28°C in an incubator for 5 h. The water conductance was measured with a conductivity meter.

Water loss rates were measured in detached leaves placed in dishes in the light at room temperature. Fresh weight was measured at set times from 0 to 7 h. The proportion of the initial fresh weight represented the water loss rate.

### DAB staining and superoxide dismutase (SOD) and hydrogen peroxidase (CAT) activity assay

H_2_O_2_ staining in leaves was performed following a previously described protocol (Hu et al., 2005). Detached leaves were infiltrated with 1 mg mL^−1^ 3,3′-diaminobenzidine (DAB) solution (contain 0.05% Tween and 10 mM Na_2_HPO_4_, pH 3.8) at 25°C for 8 h in the dark. Leaves were then immersed in 96% boiling ethanol for 15 min to disrupt the chlorophyll. The samples were transferred to fresh ethanol and photographed. The SOD and CAT were extracted from 0.2 g leaf samples by grinding them in 2 mL of 50 mM phosphate buffer (pH 7.0) at 4°C. SOD and CAT were determined using commercial assay kits purchased from Nanjing Jiancheng Bioengineering Institute (Nanjing, China), following the manufacturer's instructions. The absorbance of SOD and CAT was measured at 550 and 240 nm, respectively. All measurements were similar in all three individual replicates.

### Analysis of rice stomata by scanning electron microscopy

Leaves from wild type and *nced3* mutant plants (1 month old) were treated with water stress or 100 mM H_2_O_2_ and then immediately fixed by 3% glutaraldehyde in 0.1 M phosphate buffer (pH 7.4) at 4°C for 5 h, followed by washing twice with 0.1 M phosphate buffer for 10 min. Subsequently, these samples were fixed by 1% osmium tetroxide in 0.1 M phosphate buffer (pH 7.4) at 4°C for 2 h, washed twice with 0.1 M phosphate buffer for 10 min, dehydrated serially in 40, 50, 60, 70, 80, 90, and 100% ethanol solution for 20 min each, and then dried by critical-point dryer (Zhang et al., 2011). The samples were coated with gold and monitored by scanning electron microscopy (SU8010, Hitachi, Japan).

### Measurement of chlorophyll

Chlorophyll was extracted from 0.15 g of rice flag leaf tissue with 80% ice-cold acetone, and its content was measured according to the absorbance at 663 nm and 645 nm with a UV2400 UV/VIS spectrophotometer based on the protocol described by Lichtenthaler (1987).

### Measurement of ABA content

Leaf tissues (300 mg) were ground in liquid nitrogen. The powder was extracted with 1.5 mL of extraction solution (methanol:H_2_O:methanoic acid in a ratio of 7.9:2:0.1) and kept overnight at 4°C. The samples were centrifuged at 12,000 × *g* for 20 min at 4°C. The supernatant was collected, dried under nitrogen gas and dissolved in 2 mL of 0.1 M ammonia solution. Crude extracts were purified using a MAX column that was pre-treated with 2 mL methanol and then with 2 mL 0.1 M ammonia solution. After the supernatant was loaded onto the MAX column, the column was washed with 2 mL of 0.1 M ammonia solution and followed by 2 mL of methanol. The ABA was eluted with 4 mL of methanol containing 1% formic acid. The eluent was dried under nitrogen gas and dissolved in 0.2 mL of methanol, and then filtered through a 0.2 μm nylon membrane. [^2^H_6_]ABA was used as internal standard. ABA levels were quantified using liquid chromatography-tandem mass spectrometry (LC–MS/MS) with three biological replicates.

### Statistical analysis

All experiments were repeated in at least three biological replicates for each treatment. Data were considered statistically significant at a *P* < *0.05* using Student's *t*-test. Data are the mean ± SD from three independent experiments.

## Results

### *OsNCED3* expression pattern and protein localization

**Figure 1:**
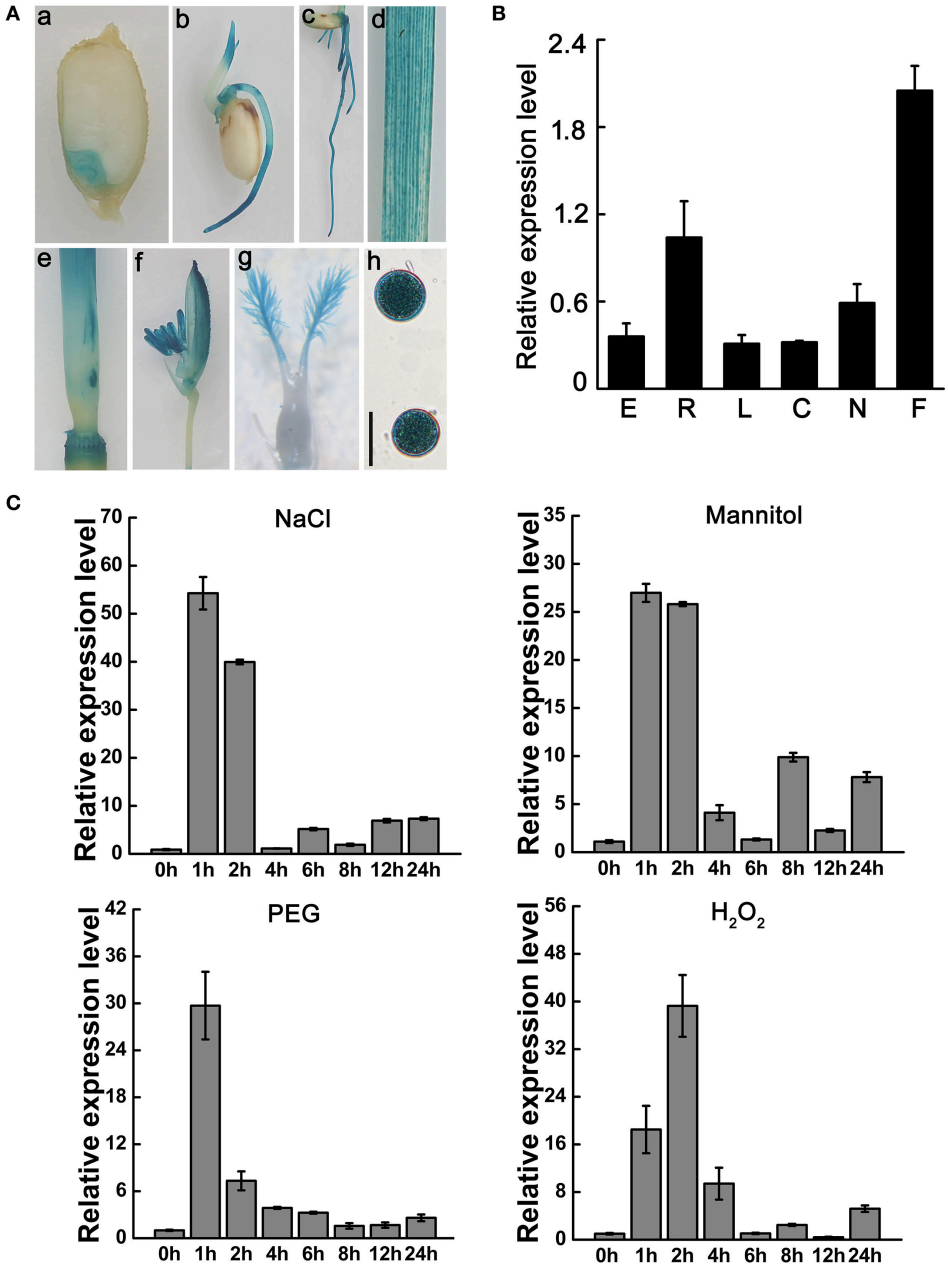
Expression analysis and subcellular localization of *OsNCED3*. **(A)** Histochemical staining of Pro_*NCED3*_-GUS transgenic plants, including mature seed (a), bud sheath (b), root (c), leaf (d), culm and node (e), flower (f), stigma (g), pollens(h). Bars = 20 μM. **(B)** Transcription level of *OsNCED3* in different organs. E(embryo), R(root), L(leaf), C(culm), N(node), F(flower). **(C)** *OsNCED3* expression in abiotic stress by qRT-PCR. The three-leaf stage seedlings were performed to 150 mM NaCl, 250 mM Mannitol, 25% PEG and 100 mM H_2_O_2_ treatment, respectively. The seedling samples were collected for *OsNCED3* expression analysis. Data are the mean ± SD for three replicates.

To observe the temporal and spatial expression patterns of *OsNCED3* in various rice plant tissues, we used the 2.27 kb upstream region from the *OsNCED3* transcriptional initiation site as the promoter to drive the reporter gene β-glucuronidase (GUS). Immunohistochemical staining of transgenic plants expressing Pro_*NCED*3_-GUS showed that *OsNCED3* was expressed in all of the tissues we tested, which included embryo, coleoptile, root, leaf, culm, node, flower, stigma, and pollen, with especially high expression in flowers and roots (Figure 1A). Quantitative RT-PCR (qRT-PCR) analysis also showed that *OsNCED3* transcripts were detected in above tissues and the highest *OsNCED3* expression in flowers and roots (Figure 1B). In addition, *OsNCED3* expression levels were significantly upregulated under NaCl, and PEG treatment for 1 h. We also found that H_2_O_2_ could rapidly induce the increase of *OsNCED3* transcript levels (Figure 1C). Additionally, GUS activity significantly increased in leaves, sheaths and roots under dehydration, NaCl and H_2_O_2_ stress, respectively (Figure S1). These results suggest that *OsNCED3* could play a role in tolerance to multiple types of abiotic stress in rice.

**Figure 2:**
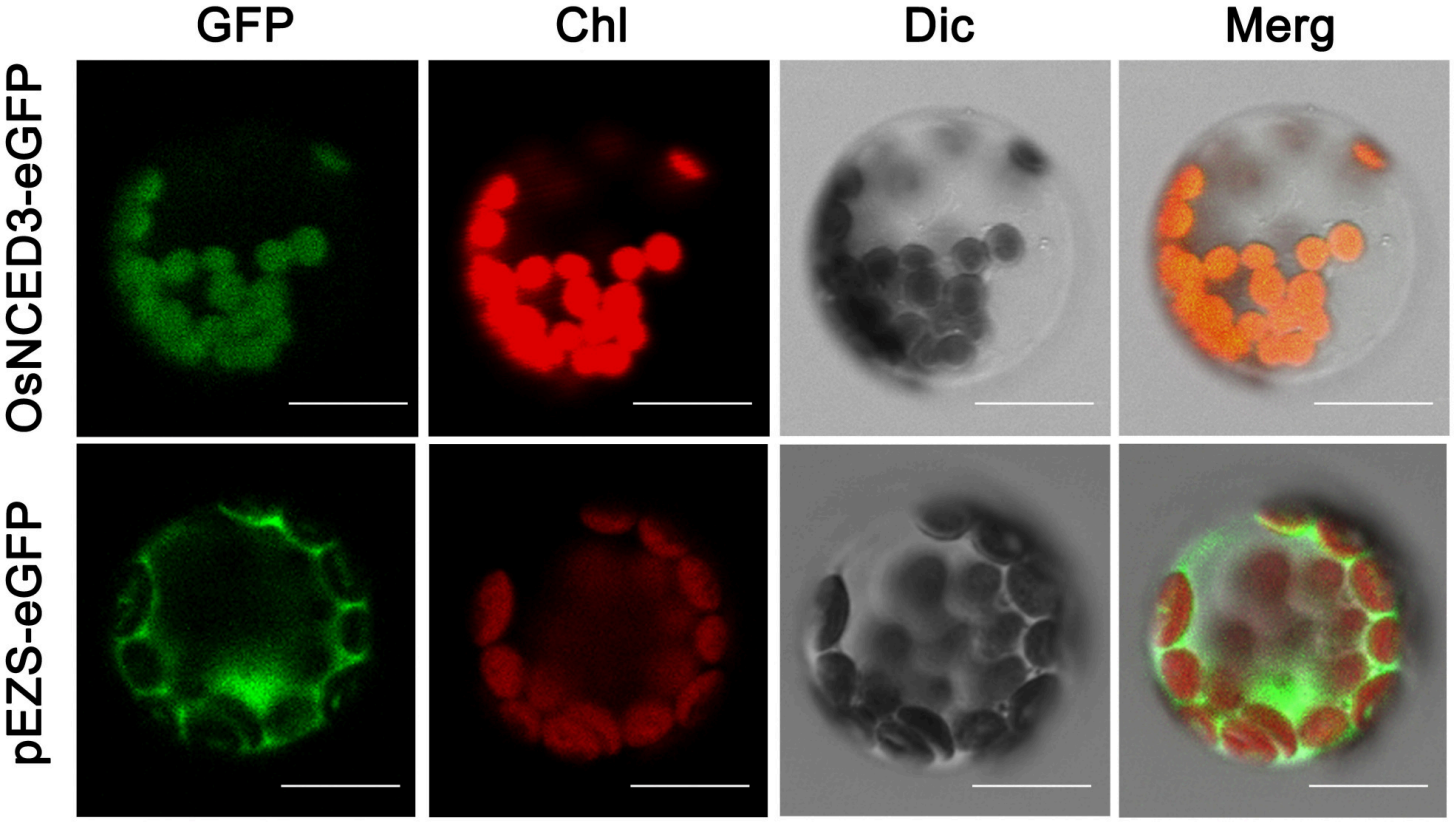
Subcellular location of OsNCED3 proteins. OsNCED3-eGFP fusion protein transiently expressed in Arabidopsis chloroplast and visualized by confocal microscopy, Bars = 20 μm.

Previous studies reported that 9-cisepoxycarotenoid dioxygenase (NCED) oxidative cleave cis-violaxanthin and cis-neoxanthin into xanthoxin was occurred in Chloroplast and other plastid (Ye et al., 2012). To identify the subcellular localization of OsNCED3, we constructed an 35S::NCED3-eGFP fusion protein and transiently transformed Arabidopsis protoplasts. Confocal microscopic visualization demonstrated that the green fluorescent signal of NCED3-eGFP co-localized with the autofluorescence of the chlorophyll, which showed that OsNCED3 was targeted to chloroplasts (Figure 2).

### *OsNCED3* regulates seed dormancy and post-germination plant growth in rice

**Figure 3:**
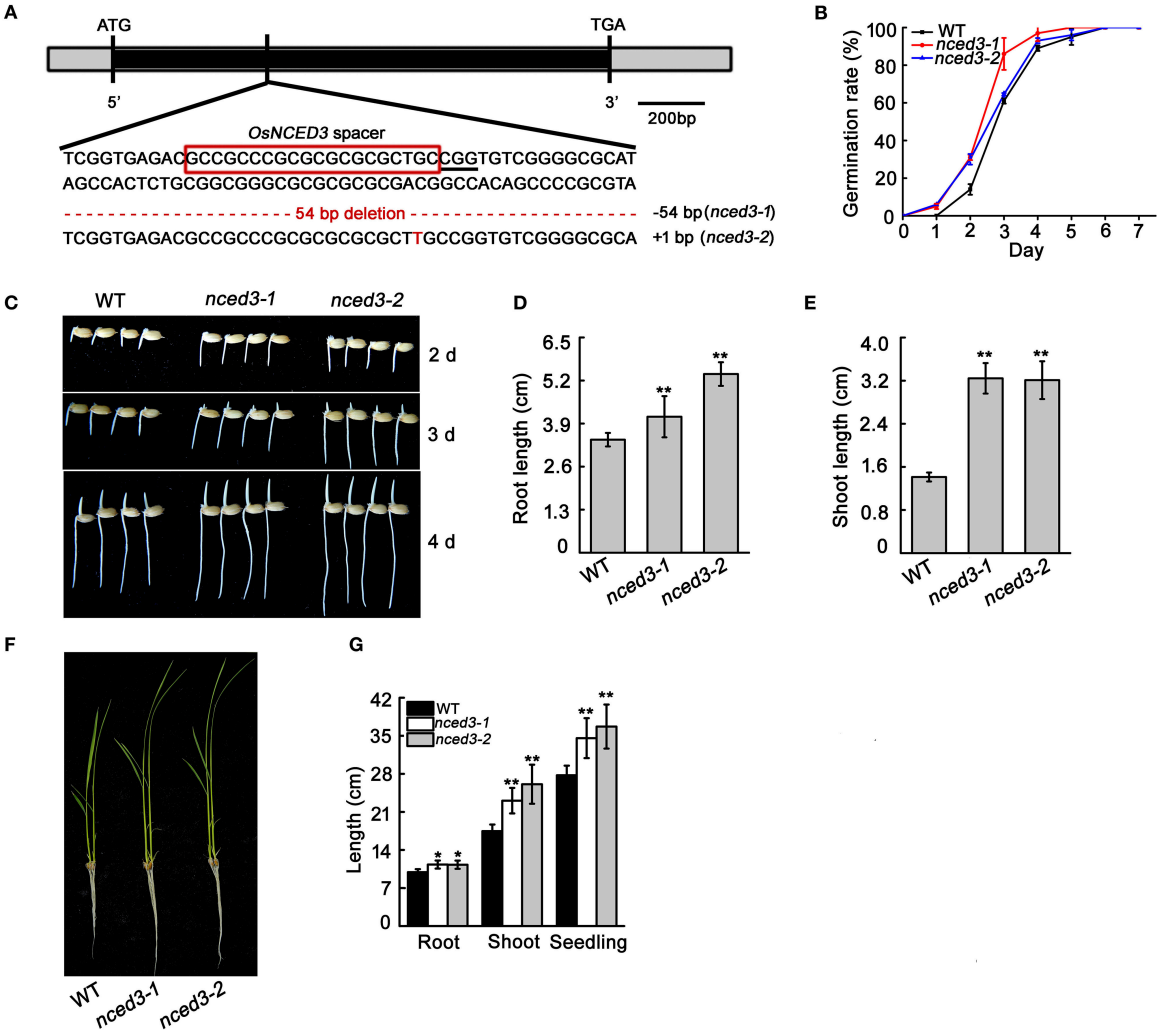
CRISPR/Cas9 induced mutation of *OsNCED3* and the phenotype of seed germination and early seedling growth in *nced3* mutants. **(A)** Targeted mutagenesis of the *OsNCED3* gene. The *OsNCED3* mutation site is shown on the gene structure. The sequence in the red box represents the *OsNCED3* spacer, and PAM is labeled by underline. The indels are shown in red dashes or letters. **(B)** Germination rate of wild type and *nced3* mutants. Data are the mean ± SD for three replicates (each replicate containing 100 seeds). **(C)** Growth state at post-germination 2 d, 3 d, and 4 d for wild type and *nced3*. **(D)** Root and **(E)** shoot length of plants post-germination 4 d. **(F)** Seedling characteristics in the three-leaf stage. **(G)** Length measurement of **(E)** seedlings. Data shown are ± SD (*n* = 14). Similar results in three individual experiments. *Asterisks* indicate statistically significant differences (Student's *t*-test; ^*^P < 0.05, ^**^P < 0.01).

*OsNCED3* is constitutively expressed in various tissues, and is significantly induced by multiple abiotic stressors. To investigate the native *OsNCED3* function in rice plant growth and abiotic stress, we used the CRISPR/Cas9 system to express pCRISPR-*NCED3* constructs, and transformed rice with *Agrobacterium tumefaciens*-mediated transformation. We obtained 35 hygromycin-resistant transgenic plants, including 32 *nced3* mutants. Sequence analysis indicated that 22.9, 3.7, and 64.8% of these 35 transgenic plants were homozygous, bi-allelic and heterozygous lines, respectively (Table S1). Two independent homozygous lines (*nced3-1* and *nced3-2*) were used for further study. The *nced3-1* mutant has a 54 bp deletion that results in an 18-amino-acid deletion in the protein, and the *nced3-2* mutant has a one-nucleotide insertion results in a frameshift mutation that promotes early termination of protein translation (Figure 3A, Figures S2, S8).

To confirm that the *OsNCED3* mutant phenotypes were not an off-target effect of CRISPR/Cas9 editing, we found seven loci with the highest ranking for off-target potential (Table S2) based on the prediction of the CRISPR-GE tool, and designed primers to amplify these regions. As shown in Figure S3, no mutations were detected at the potential off-target loci in the rice genome in these obtained mutants lines.

To investigate whether *OsNCED3* expression affects rice seed germination, the freshly harvested seeds were used for comparison of wild type and *nced3* germination rates through counting at 7 days after soaking. The germination rates of *nced3-1* and *nced3-2* mutants were slightly higher than those of the wild type (Figure 3B). In addition, the post-germinated *nced3* mutants exhibited earlier growth of roots and shoots compared to wild type seedlings, and the roots and shoots of both *nced3* mutants were significantly longer than those of the wild type (Figures 3C–E). At the three-leaf stage, *nced3-1* and *nced3-2* mutant roots and shoots were still longer than those of the wild type (Figures 3F,G). However, the mutants had no obvious differences after the tillering stage compared to the wild type when the plants were grown after this same developmental stage (data not shown). These results indicate that knock-out of *OsNCED3* may altered plant growth in seedling stage.

### Compared to wild-type rice, *nced3* mutants are more sensitive to NaCl, PEG, and oxidation stress

**Figure 4:**
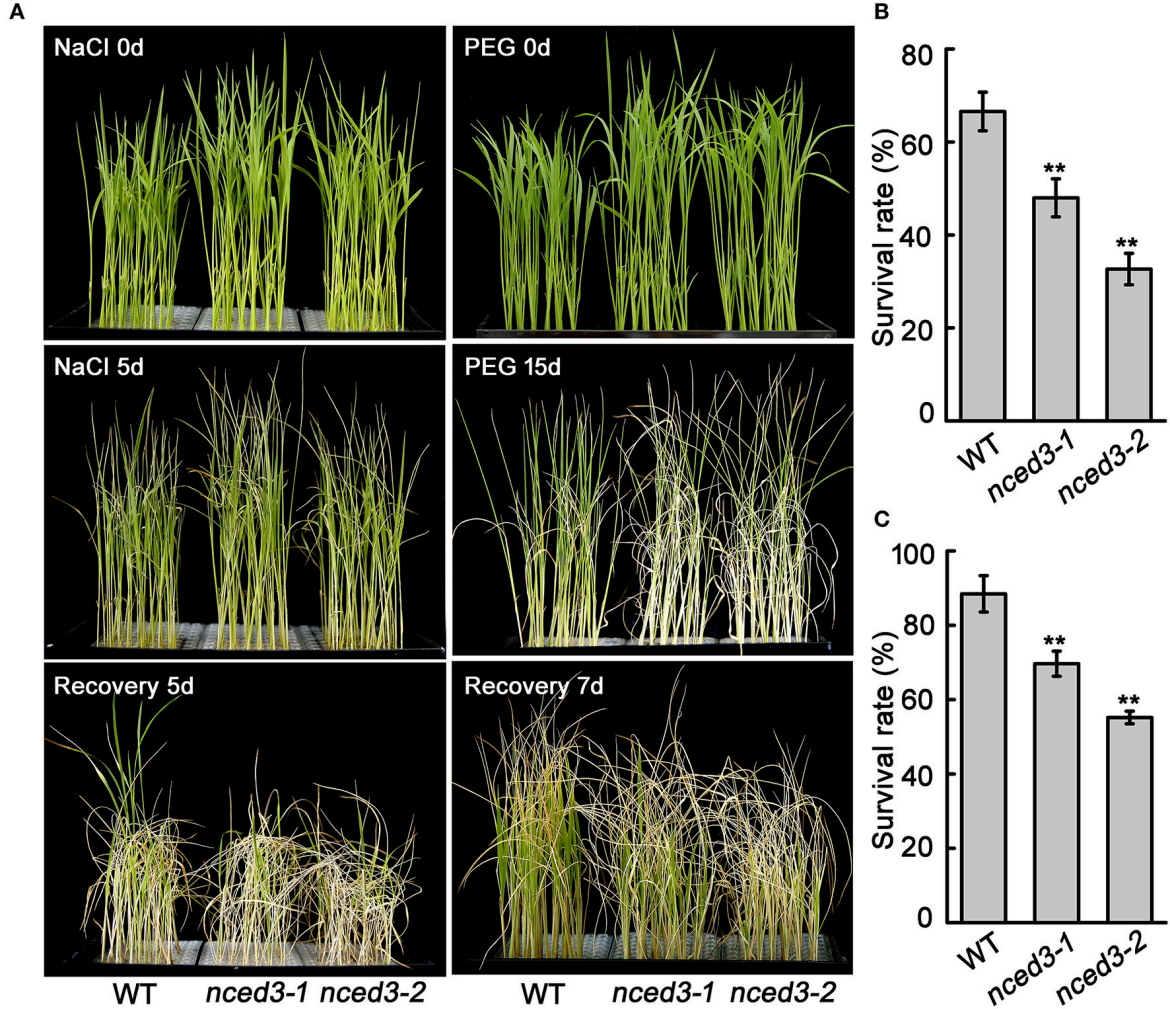
The *nced3* mutants showed decreased salt and PEG stress tolerance. **(A)** The phenotype of three-leaf stage seedlings of *nced3* mutants and wild type under NaCl and PEG stress. **(B,C)** Statistics for survival rate after salt and PEG stress. The number of surviving plants as a proportion of the total plants is shown. Data shown are ± SD from three independent replicates. *Asterisks* indicate statistically significant differences (Student's *t*-test; ^**^P < 0.01).

Our qRT-PCR analysis showed that *OsNCED3* expression was noticeably induced by NaCl and PEG stress. Therefore, we treated 2-week-old *nced3* mutant and wild type seedlings with Hoagland's nutrient solution that contained 150 mM NaCl or 25% PEG. After 5 days of NaCl stress, more leaves of the two *nced3* mutants had withered compared to the wild type plants. After the high-salt treatment, the plants were transferred to normal nutrient conditions to recover for 5 d. The recovery was 48% for *nced3-1* and 32.7% for *nced3-2*, which was lower than the 66.6% observed for the wild type (Figures 4A,B). Furthermore, 2-week-old *nced3* mutants and wild type seedlings were subjected to 25% PEG treatments for 15 d, after which most of the *nced3* mutants leaves withered, whereas the wild type showed slightly response. The seedlings were then transferred to normal conditions and allowed to recover for 7 d, but only 69.7% and 55.2% of the *nced3-1* and *nced3-2* seedlings survived, respectively, which were significantly lower than the 88.4% survival observed for the wild type (Figures 4A,C). These results indicate that the *nced3* mutants had a decreased capacity for high salinity and osmotic stress tolerance.

**Figure 5:**
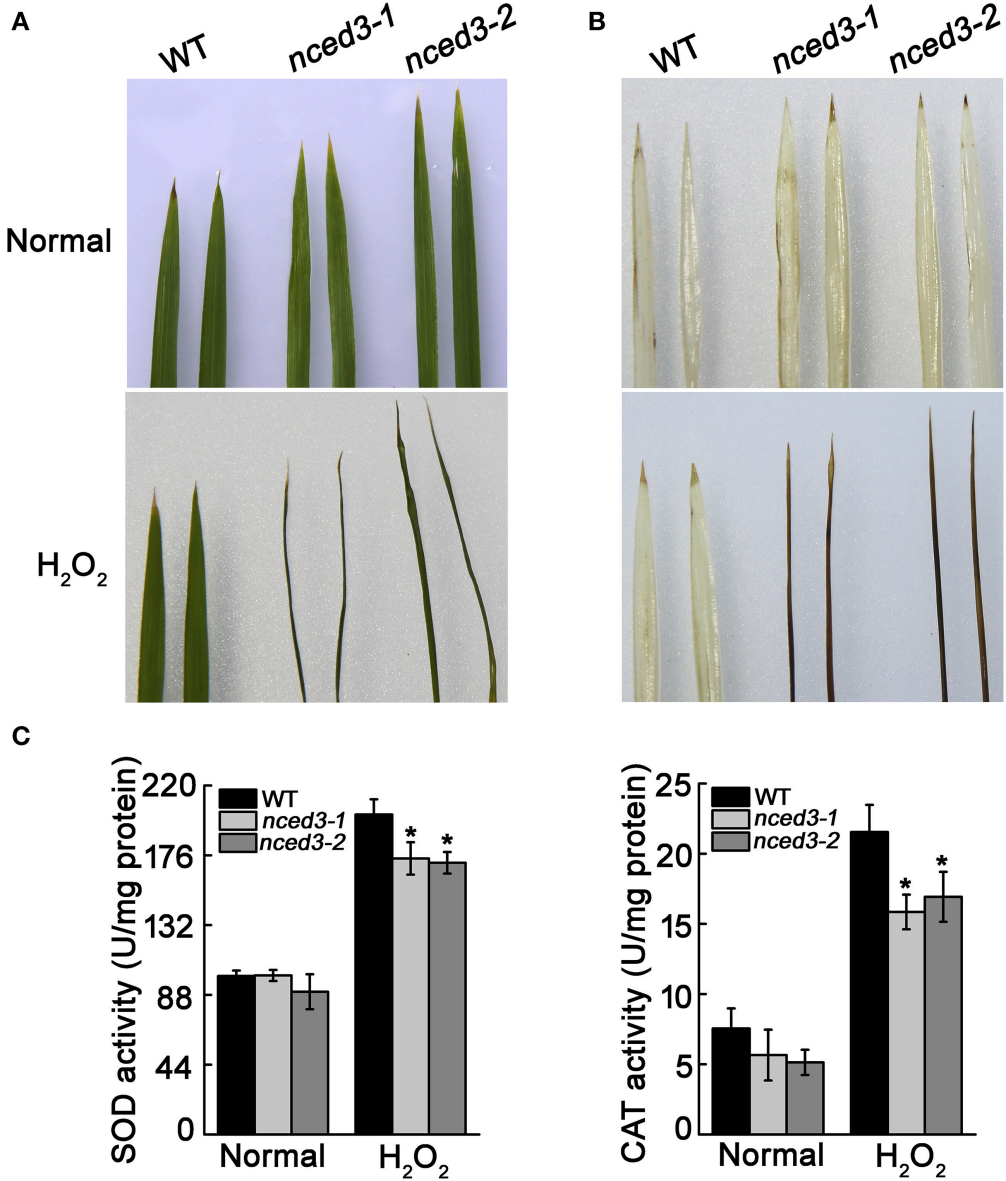
Analysis of H_2_O_2_ accumulation and oxidative enzymes under H_2_O_2_ stress condition. **(A)** Phenotype of *nced3* and wild type leaves after 100 mM H_2_O_2_ treatment for 2 d. **(B)** DAB staining of H_2_O_2_ accumulation. Three independent replicates were performed. **(C)** SOD and CAT activities of *nced3* and wild type three-leaf-stage seedlings under 100 mM H_2_O_2_ treatment for 24 h. Data shown are ± SD from three independent replicates. *Asterisks* indicate statistically significant differences (Student's *t*-test; ^*^P < 0.05).

*OsNCED3* also responded to H_2_O_2_ treatment. To examine whether *OsNCED3* confers ROS tolerance, *nced3* mutants and wild type seedlings were subjected to H_2_O_2_ treatment for 2 d at the three-leaf stage. Whereas almost all of the *nced3* leaves had wilted under these conditions, wild type leaves were generally unaffected (Figure 5A). Using DAB staining, we detected no differences in the untreated leaves of *nced3-1, nced3-2* and wild type rice, whereas the leaves of *nced3-1* and *nced3-2* plants had dark brown discoloration compared to wild type leaves, which only had a few brown regions at the leaf apexes (Figure 5B). These observations suggest that *OsNCED3* loss-of-function mutant plants were susceptible to H_2_O_2_ stress. H_2_O_2_ is a vital stress signaling molecule in plants, and can increase the superoxide dismutase (SOD) and hydrogen peroxidase (CAT) activity in leaves (Hu et al., 2005). To determine whether *nced3* mutants had altered antioxidant enzyme activity during H_2_O_2_ treatment, SOD and CAT activity was measured. The activity of these two antioxidant enzymes in *nced3* mutants and wild type plants were enhanced during H_2_O_2_ treatment as compared to control conditions, but the SOD and CAT activity of *nced3* mutants was lower than that of wild type seedlings under H_2_O_2_ stress (Figure 5C). These results indicate that *OsNCED3* could promote antioxidant enzyme activity when plants are exposed to oxidative damage.

### *nced3* mutant rice had decreased water stress tolerance and stomata closure

**Figure 6:**
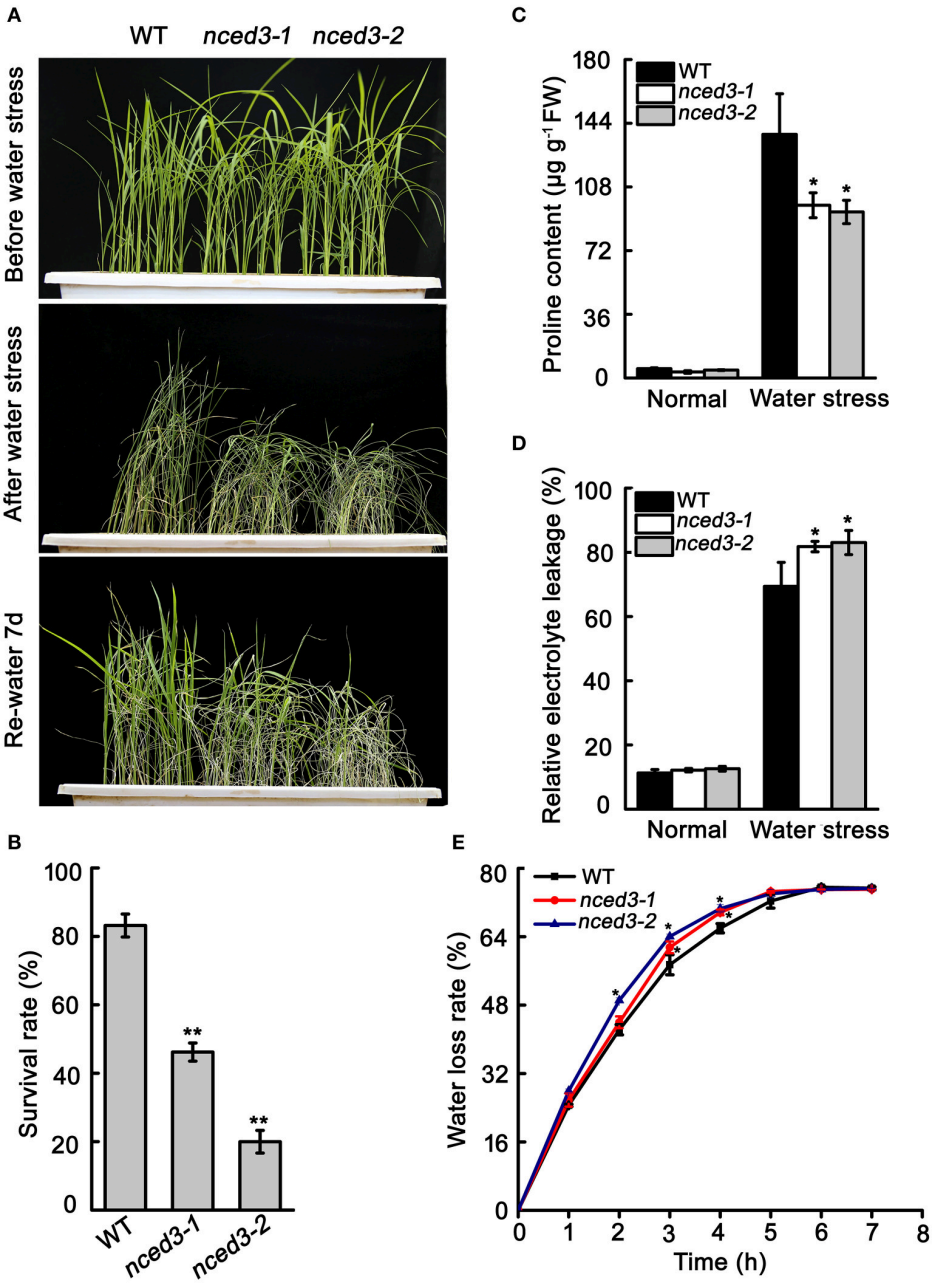
Water stress tolerance of *nced3* seedlings. **(A)** Phenotype of *nced3* in water stress. **(B)** Survival rate after drought stress. The number of surviving plants as a proportion of the total plants is shown. **(C, D)** Proline and relative electrolyte leakage determination after water treatment. Data shown are ± SD from three independent replicates. **(E)** Measurement of water loss rate in wild type, *nced3-1*, and *nced3-2* seedlings. Values are the mean ± SD (*n* = 6 plants). Similar results were obtained from three independent replicates. *Asterisks* indicate statistically significant differences (Student's *t*-test; ^*^P < 0.05, ^**^P < 0.01).

The *nced3* mutants and wild type seedlings were grown to the three-leaf stage in soil before watering was withheld for 15 d, and then watering was resumed for 7 d. Approximately 83.2% of the wild type plants recovered from this water stress treatment, but only 40.2 and 20% of the *nced3-1* and *nced3-2* plants recovered, respectively (Figures 6A,B). The water stress sensitivity of the *nced3* mutants was also tested at the reproductive stage. We observed that 76.7 and 85.9% of the *nced3-1* and *nced3-2* mutant leaves rolled, respectively, which was more than was observed in the wild type (35.3%) under water stress (Figures S5A–C). After water stress, the seed set rates of *nced3-1* and *nced3-2* mutants were only 55.8 and 47.7%, which was distinctly lower than the 70.7% seed set rate measured in the wild type (Figures S5D,E).

Given that the altered physiological mechanism possibly responsible for water stress adaption. we examined proline content and relative electrolyte leakage in leaf tissues. Under normal conditions, these two factors were very low, and there were no differences between the wild type and *nced3* mutants. After 10 d of water stress, proline content in leaves significantly increased in the wild type and the two *nced3* mutants; however, the *nced3* mutants leaves accumulated less proline than the wild type (Figure 6C). At the same time, relative electrolyte leakage was greater in *nced3* mutants than in the wild type under drought stress (Figure 6D). We also evaluated water loss avoidance in *nced3* and wild type plants by water loss rate analysis. The detached leaves of *nced3* mutants lost water faster than those of the wild type (Figure 6E). These results indicate that *OsNCED3* plays a vital role in water stress tolerance in rice.

**Figure 7:**
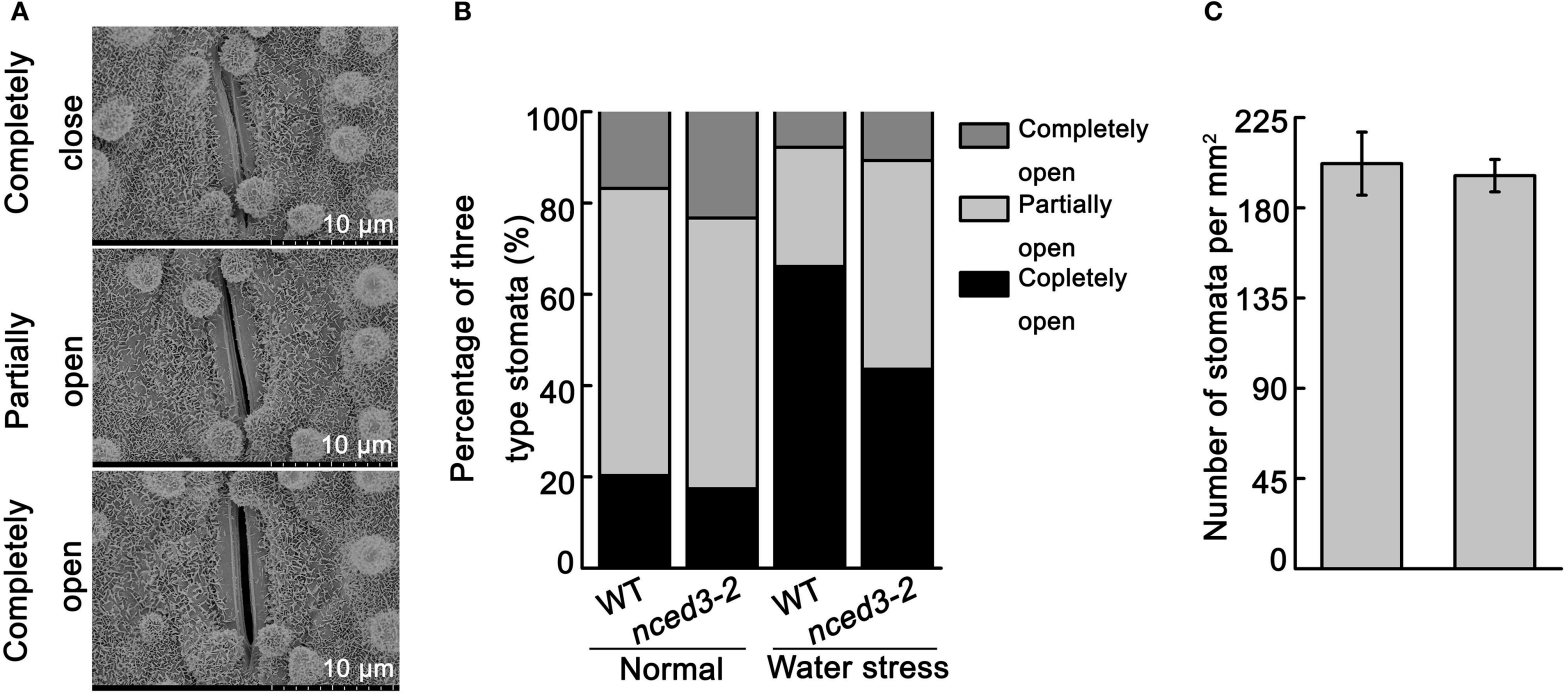
Stomata closure of *nced3* mutant under water stress. **(A)** Scanning electron microscopy images of three levels of stomatal apertures, Bars = 10 μm. **(B)** The percentage of three levels of stomatal apertures in *nced3-2* mutant and wild type plants were calculated under normal and water stress condition (*n* = 105 stomata for wild type; *n* = 98 stomata for *nced3-2* mutant). **(C)** Stomata numbers per mm^2^ were counted in middle leaves from *nced3-2* mutant and wild type plants, respectively. Data shown are ± SD from there independent replicate.

To further analyze that the water loss rate in *nced3* mutants were more faster than wild type. We investigated the stomatal aperture and density in *nced3-2* mutant and wild type plants. Under normal conditions, the percentages of completely open stomata in the *nced3-2* mutant plants were little higher than wild type plants, but other two-type stomata have no difference between *nced3-2* mutant and wild type (Figures 7A,B). However, under water stress conditions, 66.1% of stomata were completely closed in wild type plants, while only 43.6% were completely closed in *nced3-2* mutant plants. Additionally, only 26.1% of stomata were partially open in wild type plants, but 45.7% were partially open in *nced3-2* mutant plants. Furthermore, 10.7% of stomata were completely open in *nced3-2* mutant plants, and no significant difference was observed compared to the 7.8% of completely open stomata in wild type plants (Figure 7A,B). We also observed stomatal density, and confirmed that there were no obviously differences between *nced3-2* mutant and wild type plants (Figure 7C). These results indicate that *OsNCED3* controls stomata movement under water stress conditions.

### *OsNCED3* affects ABA accumulation and ABA-related gene expression

**Figure 8:**
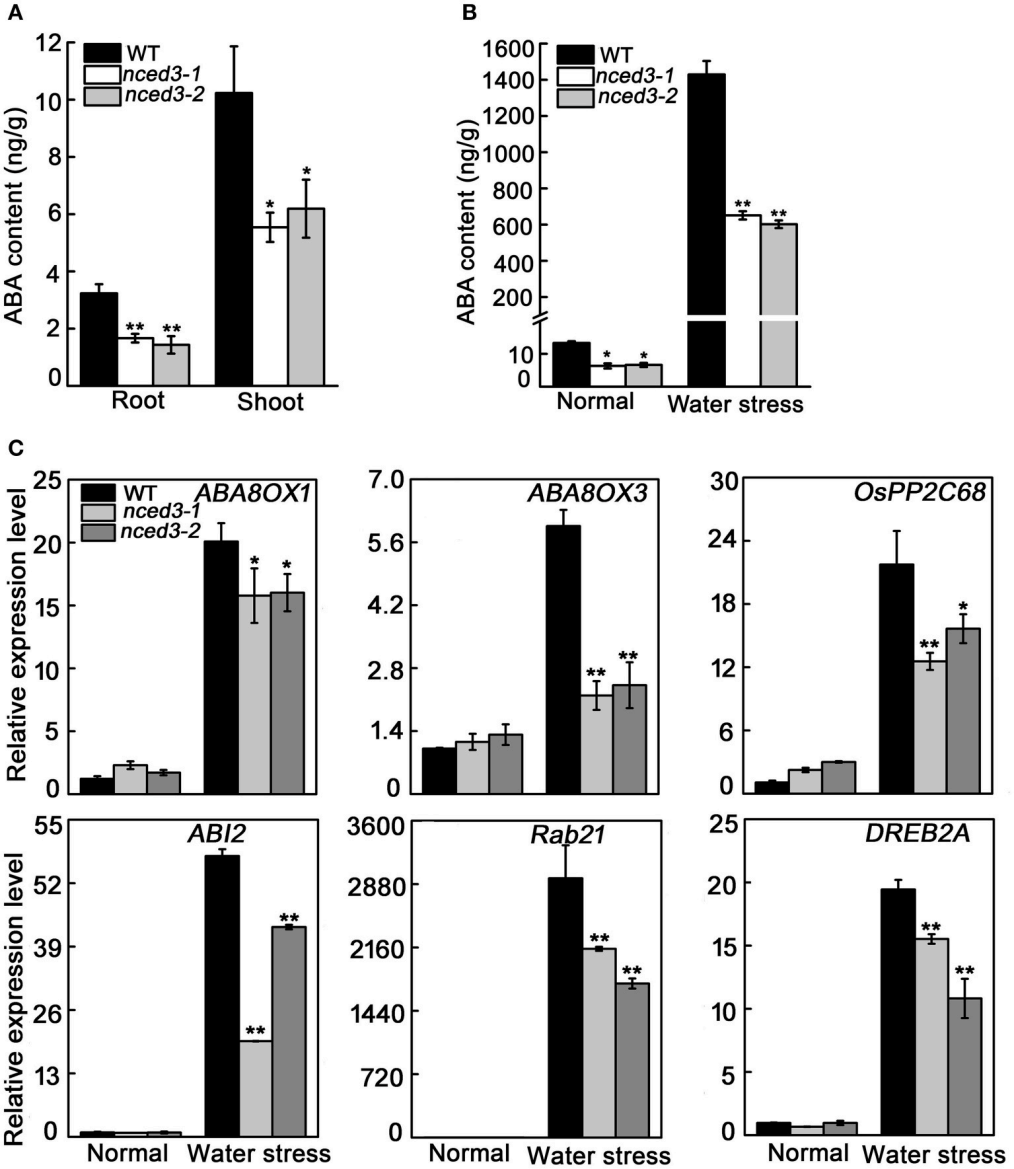
ABA accumulation and ABA-related gene expression in *nced3* mutants. **(A)** The shoot and roots ABA content in normal condition, **(B)** The leaf ABA content under water stress, **(C)** The leaf ABA-related genes expression in wild type and *nced3* mutants three-leaf stage seedlings under water stress for 5 d. Data shown are ± SD from there independent replicate. *Asterisks* indicate statistically significant differences (Student's *t*-test; ^*^P < 0.05, ^**^P < 0.01).

ABA was well described as regulates plant growth and enhances water stress adaption, and NCED catalyzes the key regulatory step in ABA biosynthesis (Ng et al., 2014). Hence, we measured the ABA content of shoots and roots at the three-leaf stage, and found that the ABA levels in the shoots of mutants (5.5 ng/g for *nced3-*1 and 6.2 ng/g for *nced3-2*) and roots of mutants (1.7 ng/g for *nced3-1* and 1.4 ng/g for *nced3-2*) were both noticeably lower than that in wild type plants (10.2 ng/g in shoots and 3.2 ng/g in roots) (Figure 8A). Therefore, *nced3* mutants had longer shoots and roots than wild type plants, possibly due to a decrease in ABA content. Moreover, the ABA levels in the leaves of *nced3* mutants and wild type were also investigated under normal and water stressed growth conditions. Under normal conditions, ABA levels in the *nced3* mutants (6.3 ng/g for *nced3-1* and 6.6 ng/g for *nced3-2*)were approximately 50% of that in wild type (13.3 ng/g). After the water stress treatment, ABA content in *nced3* and wild type seedlings significantly increased, but the wild type (1429.4 ng/g) ABA content was 2.3 times higher than *nced3* mutants (650.9 ng/g for *nced3-1* and 601.9 ng/g for *nced3-2*) (Figure 8B). Therefore, the increased water stress sensitivity in the *nced3* mutant seedlings correlates with the mutation in *OsNCED3*, which could reduce endogenous ABA biosynthesis.

We further determined the transcript levels of ABA catabolic genes and ABA signaling pathway genes under water stress. Consistent with the increase in ABA levels after water stress treatment, expression of *OsABA8ox1, OsABA8ox3, OsPP2C68, OsABI2, OsRab21*, and *OsDREB2A* genes were noticeably induced in all plants, but *nced3* mutant seedlings had less of an increase compared to the expression levels measured in wild type seedlings (Figure 8C). These results suggest that *OsNCED3* plays an important role in ABA biosynthesis, and indirectly up-regulates ABA-related gene transcription under water stressed growth conditions.

### Overexpression of *OsNCED3* enhanced NaCl and water stress tolerance and increased endogenous ABA content

**Figure 9:**
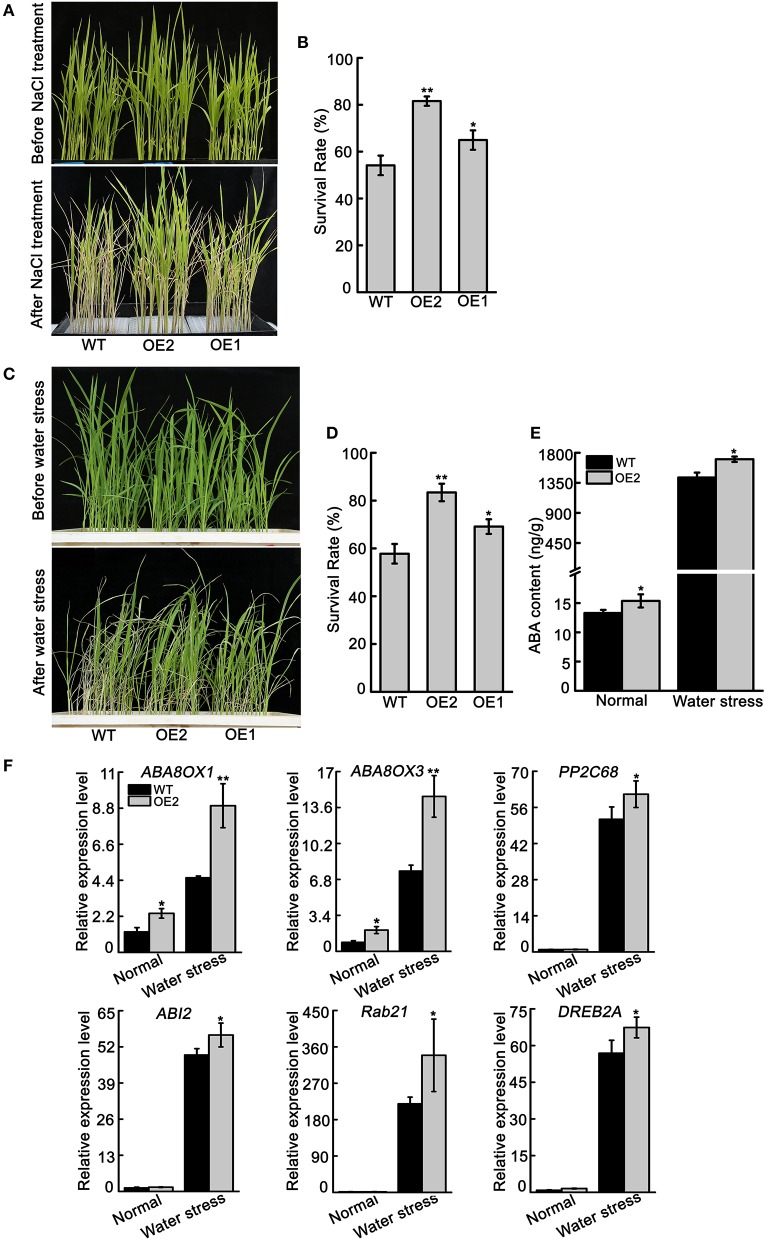
The phenotype of *OsNCED3*-overexpressing plants and ABA content and ABA-related genes transcript levels. **(A)** The phenotype of three-leaf stage seedlings of *OsNCED3*-overexpressing and wild type under 150 mM NaCl stress. **(B)** Statistics for survival rate after salt stress. The number of surviving plants as a proportion of the total plants is shown. **(C)** The phenotype of three-leaf stage seedlings of *OsNCED3*-overexpressing and wild type under water stress. **(D)** Statistics for survival rate after water stress. **(E)** ABA content and **(F)** ABA-related genes expression in *OsNCED3*-overexpressing and wild type three-leaf stage seedlings under water stress for 5 d. Data shown are ± SD from there independent replicate. *Asterisks* indicate statistically significant differences (Student's *t*-test; ^*^P < 0.05, ^**^P < 0.01).

To confirm the function of *OsNCED3* in response to NaCl and water stress, transgenic lines (OE1, OE2) of *OsNCED3*-overexpressing plants were obtained (Figure S6). The growth of *OsNCED3*-overexpressing plants were similar to wild type plants. However, *OsNCED3*-overexpressing plants were more tolerant to high salinity stress compared to wild type plants (Figure 9A). The survival rate of OE1 and OE2 was 64.9 and 81.6%, respectively, but the survival rate of wild type was only 54.1% (Figure 9B). Additionally, the *OsNCED3*-overexpressing seedlings and wild type seedlings were grown in soil and withholding water for water stress analysis. After withholding water for 18 d and re-watering, the *OsNCED3*-overexpressing plants had enhanced tolerance to water stress compared to with wild type (Figure 9C). Approximately 57.8% of the wild type plants recovered from water stress conditions, but 69.1 and 84.3% of the OE1 and OE2 transgenic plants recovered, respectively (Figure 9D).

In addition, the endogenous ABA content was measured in the leaves of *OsNCED3-*overexpressing and wild type plants under normal and water stress conditions. Under normal conditions, the ABA content in *OsNCED3*-overexpressing plants (15.4 ng/g) was higher than in wild type plants (13.3 ng/g). After water stress for 10 d, the amount of endogenous ABA accumulated in both *OsNCED3*-overexpressing and wild type plants, but the ABA content in *OsNCED3*-overexpressing plants (1702.8 ng/g) was much higher than in wild type plants (1429.4 ng/g) (Figure 9E). Consistently, the transcript levels of ABA related genes were significantly induced in *OsNCED3*-overexpressing and wild type plants under water stress conditions, but the expression levels were much higher in *OsNCED3*-overexpressing plants than in wild type plants (Figure 9F). Interestingly, the transcript levels of *ABA8OX1* and *ABA8OX3* were also higher in *OsNCED3*-overexpressioning plants than in wild type plants under normal condition, but other genes were not obviously different between *OsNCED3*-overexpressing and wild type plants. These results further indicate that *OsNCED3* could promote endogenous ABA accumulation and enhance water stress tolerance.

### *OsNCED3* promotes leaf senescence in detached rice leaves

**Figure 10:**
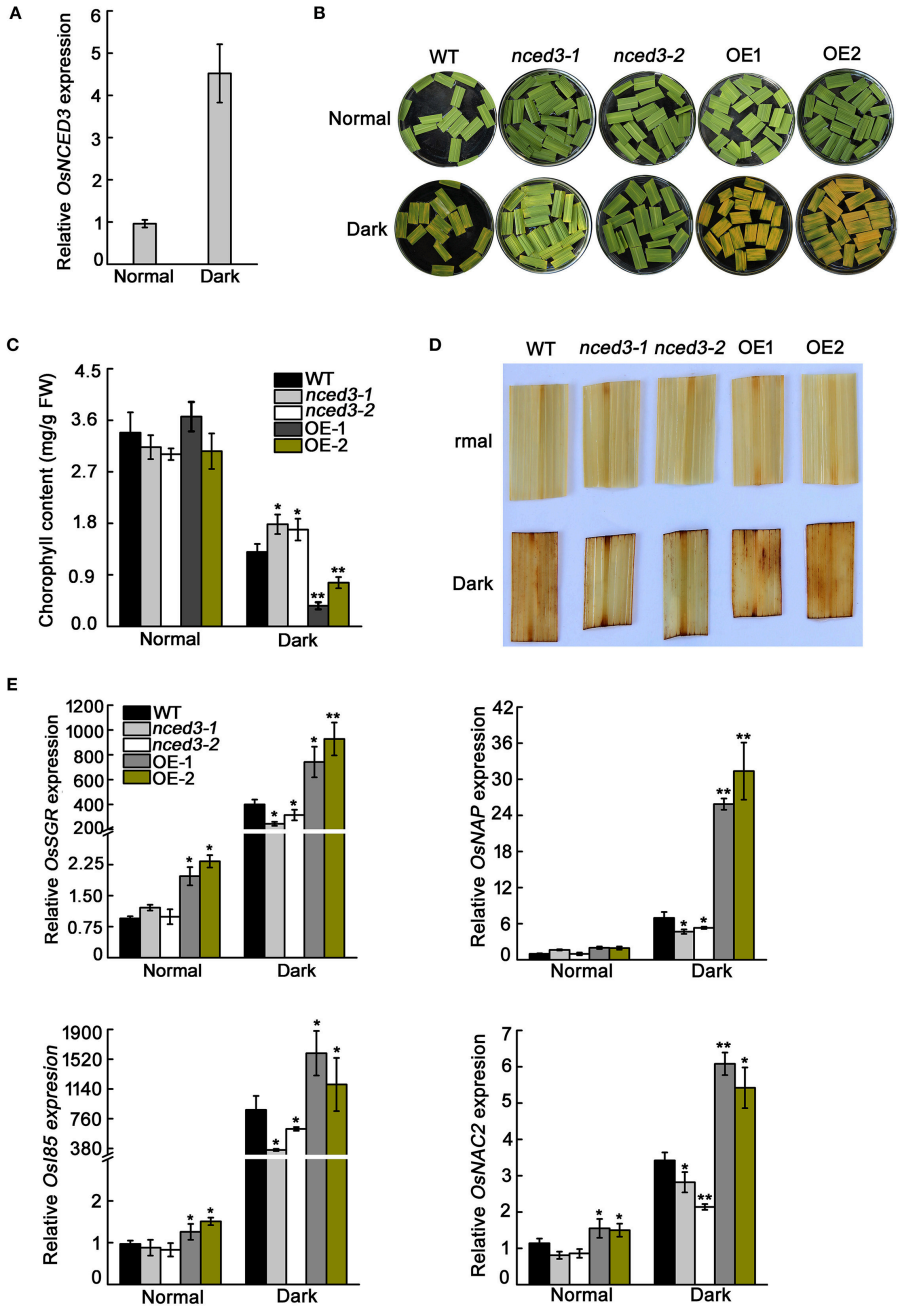
Dark-induced leaf senescence phenotype of leaf sections in *nced3* mutant and *OsNCED3-*overexpressing plants. **(A)** Quantitative real-time PCR analysis of *OsNCED3* expression after 3-day dark treatment. Data are the mean ± SD for three replicates. **(B)** Leaf senescence phenotype of 8-week-old *nced3-1,2* and OE-1,2 plants after 3-day dark treatment. **(C)** Measurement of chlorophyll content in the 8-week-old *nced3-1,2* and OE-1,2 leaves after dark treatment. **(D)** DAB staining of detached *nced3-1,2* and OE-1,2 leaves after dark treatment. **(E)** Relative expression of *OsSGR, OsNAP, OsI85*, and *OsNAC2* genes after 3-day dark treatment in detached *nced3-1*,*2* and OE-1,2 leaves. Data shown are ± SD from three independent replicates. *Asterisks* indicate statistically significant differences (Student's *t*-test; ^*^P < 0.05, ^**^P < 0.01).

ABA is a positive regulator of leaf senescence (Lee et al., 2011). Dark treatment is an effective method to accelerate leaf senescence and ABA biosynthesis (Mao et al., 2017). *OsNCED3* is a pivotal ABA biosynthesis gene and its transcription level in leaves is strongly induced in dark treatment compared to normal light conditions (Figure 10A). Consistent with the qRT-PCR results, β-glucuronidase activity was more abundant in leaves after 3 d of dark treatment than in leaves under control condition (Figure S7). The segments of leaves from *nced3* mutants displayed minor leaf senescence in darkness, whereas *OsNCED3*-overexpressing in the OE1 and OE2 transgenic plants had a significant leaf senescence phenotype when compared with wild type plants under the same conditions (Figure 10B). Darkness led to significant loss of chlorophyll content in OE1 and OE2 detached leaves, but the decrease in chlorophyll content in *nced3* mutants was significantly less than that in the wild type after 3 d of dark treatment (Figure 10C).

We further examined the H_2_O_2_ and senescence marker genes expression levels in darkness-treated leaves. The OE1 and OE2 detached leaves accumulated significantly higher amounts of H_2_O_2_ than wild type by DAB staining, whereas the H_2_O_2_ accumulation in *nced3* mutants was lower than in the wild type (Figure 10D). As expected, transcription levels of the senescence marker genes *OsSGR, OsNAP, OsI85*, and *OsNAC2* in OE1 and OE2 dark-treated leaves were significantly higher compared to those of the wild type, whereas these genes were showed much lower expression in the *nced3* lines than in the wild type (Figure 10E). Thus, *OsNCED3* is a positive regulator of ABA biosynthesis, which is related to plant leaf senescence.

## Discussion

ABA is an essential hormone for seed dormancy, stomata aperture, plant development, and abiotic stress tolerance. The levels of ABA in plants is regulated by its *de novo* biosynthesis. NCED is the critical rate-limiting enzyme in the ABA biosynthetic pathway. *OsNCED3* is a member of *NCED* gene family in rice, and although the partial function of *OsNCED3* has been reported, its native functional characteristics in rice is still unclear. Here, we studied the functional characteristics of *OsNCED3* by successfully creating *nced3* mutant lines and *OsNCED3*-overexpressing plants.

Increased levels of ABA or inhibition of ABA degradation can maintain seed dormancy in plants (Martinez-Andujar et al., 2011). The *OsNCED3* ortholog in maize, *VP14*, is specifically expressed in mature embryos, and its mutation resulted in *nced3* mutant lines that displayed early seed germination (Figure 3B). These phenotypes were similar to the maize *vp14* mutant (Schwartz et al., 2003). Other ABA biosynthesis-deficient mutants, including *Osaba1* in rice (Agrawal et al., 2001) and *Atnced6* and *Atnced9* in Arabidopsis, contribute to seed germination (Lefebvre et al., 2006). Overexpression of ABA biosynthetic pathway genes, including *NpZEP* (Frey et al., 2006), *TaNCED2* (Son et al., 2016), *LeNCED1* (Tung et al., 2008), and *GINCED1* (Zhu et al., 2007), occurs in many higher plants, and promotes seed dormancy. Thus, *OsNCED3* could regulate ABA biosynthesis enzymes required for seed dormancy through increasing the ABA content in seeds.

Endogenous ABA acts as a dual modulator for plant growth and development. When excessive ABA accumulates in plant organs, it can inhibit plant development, but low endogenous ABA levels can sometimes promote plant growth (Cheng et al., 2002). The *nced3* mutant lines reduced ABA content in seedlings (Figure 8A), and the shoots and roots were both significantly longer than that in the wild type (Figures 3C–G), but the growth of *OsNCED3*-overexpressing lines were not different from that of wild type, although ABA content was higher in *OsNCED3*-overexpressing lines (Figure S6 and Figure 9E). However, ectopic expression of *OsNCED3* in Arabidopsis resulted in smaller and rounder leaves and midveins compared to wild type plants (Hwang et al., 2010). It is possible that *OsNCED3* regulates plant development in rice or Arabidopsis through different mechanisms. Furthermore, the Arabidopsis ABA-deficient *aba1* and *aba2* mutants are wilted and small relative to wild type, and exogenous ABA could promote plant growth in *aba1* and *aba2* mutant plants (Barrero et al., 2005; Lin et al., 2007). The rice *nced3* mutants displayed a different phenotype, compared to Arabidopsis ABA-deficient mutants. The higher stomata aperture in *nced3* mutant possibly altered transpiration and photosynthesis, thus produced this diverse phenotype. Our results indicate that the precise modulation of ABA levels plays an important and positive role in plant growth regulation under normal environmental conditions.

The *NCED* genes contribute to increased ABA levels, which promotes abiotic stress tolerance in plants (Bang et al., 2013). Our study shows that expression of *OsNCED3* was significantly induced by NaCl and osmotic stress (Figure 1C). Similarly, the *nced3* mutants were hypersensitive to NaCl and PEG stress (Figure 4), and exhibited a significant decrease in water stress tolerance (Figure 6A). Moreover, *OsNCED3*-overexpressing plants had enhanced NaCl and water stress tolerance (Figures 9A–D). Several other *NCED* genes, including *VP14* (Tan et al., 1997), *AtNCED3* (Frey et al., 2012), *CsNCED3* (Pedrosa et al., 2017), *SgNCED* (Yang and Guo, 2007), and *LeNCED1* (Tung et al., 2008) were previously reported to alter water stress sensitivity if expression was inhibited or overexpressed in plants. Under water stress conditions, the plants underwent a physiological transformation to adapt to the severe environmental conditions. Proline is an osmolyte that can facilitate adaptation to water deficient conditions (Voetberg and Sharp, 1991), and electrolyte leakage is a physiological index that indicates ability of plants to resist to water stress (Jiang et al., 2016). Our results suggest that the *nced3* mutants were sensitive to water stress, which can possibly be attributed to the fact that they had a low proline content and high relative electrolyte leakage (Figures 5C,D). The results were further confirmed by the enhanced water stress tolerance of *OsNCED3*-overexpresing plants (Figure 9C). Similar observations were observed in *BnNCED3* transgenic plants (Xu and Cai, 2017). Furthermore, ABA reduce water loss by regulating stomatal closure in order to acclimate to water stress (Finkelstein, 2013). The percentage of completely close stomata in *nced3* mutant is much lower than wild type plants under water stress. This is consistent with previous studies found that *sapk2* mutants affect ABA-dependent stomatal movement (Lou et al., 2017). This result indicates that *OsNCED3* positively regulates water stress tolerance by reducing water loss through facilitating stomata closure. Additionally, the ABA content in *nced3* mutants was much lower than that measured in wild type plants under water stress conditions (Figure 8B), but *OsNCED3*-overexpresing plants accumulated higher ABA levels compared to wild type plants (Figure 9E) which is consistent with the observation that *AtNCED3* and *AtNCED5* regulate ABA accumulation under water stress (Frey et al., 2012). On the other hand, the transcription of ABA catabolic genes such as *OsABA8ox1* and *OsABA8ox3* (Shi et al., 2015), and signaling pathway genes such as *OsPP2C68, OsABI2, OsRab21*, and *OsDREB2A* is significantly induced to improve water stress adaptation in rice (Cui et al., 2011; Chen et al., 2014). Our study showed that the expression levels of these genes were significantly lower in *nced3* mutants, but obviously higher in *OsNCED3*-overexprssing plants compared with wild type plants under water stress. These results indicate that *OsNCED3* is responsible for ABA accumulation, and may be indirectly regulating the expression of ABA-dependent water stress-related genes under water stress.

Previous study showed that water stress-induced ABA triggered an elevation in ROS in plants to regulate stomata aperture (Yao et al., 2013); Under exogenous H_2_O_2_ treatment, the percentage of completely closed stomata in *nced3* mutant was much lower than wild type plants (Figure S4), which is consistent with the observation that *nced3* mutants had lower tolerance to oxidation stress than the wild type plants. Ordinarily, excessive H_2_O_2_ stimulates the dual ABA induced ROS-scavenging system to resist oxidation damage (Desikan et al., 2004; Ozfidan et al., 2012). The activity of SOD and CAT in wild type leaves was higher than the enzymatic activity measured in *nced3* mutant leaves under exogenous H_2_O_2_ treatment (Figures 5C,D). This is similar to the observation that reduced expression of the *OsABA8ox3* gene resulted in decreased antioxidant enzyme activity, and increased oxidative damage (Cai et al., 2015). The results for the ABA deficiency in the *nced3* mutants indicate that these plants normally avoid oxidative damage by increasing ABA biosynthesis through a pathway that requires *OsNCED3* gene function during oxidative stress.

ABA is a positive regulator for leaf senescence. Previous studies have shown that *OsNAC2* directly binds to the promoters of the ABA metabolism-related genes *OsNCED3, OsZEP1*, and *OsABA8ox1*, which control the production of ABA in plants, and regulate ABA-dependent leaf senescence (Mao et al., 2017). Our study indicates that the expression of *OsNCED3* was significantly induced by dark treatment, and leaf sections of *nced3* mutants had greener leaves in the dark, but *OsNCED3*-overexpressing leaf sections quickly turned yellow in the dark (Figure 10A,B). Furthermore, the transcription factors *OsNAC2* and *OsNAP* participated in the aging process dependent on ABA biosynthesis and the leaf-senescence marker genes *OsI85* and *OsSGR* (Chen et al., 2014; Mao et al., 2017). The transcription levels of these genes in dark-treated leaves from the *OsNCED3*-overexpressing lines were notably higher than those in the *nced3* mutants (Figure 10E). This is similar to the observation that overexpression of *BnNCED3* in Arabidopsis promotes leaf senescence (Xu and Cai, 2017). Consequently, our results further confirm that *OsNCED3* acts as a key regulator of ABA biosynthesis and links ABA production with leaf senescence.

Collectively, our observations found that *OsNCED3* controls ABA levels in rice to ensure seed dormancy, growth and stomatal aperture, while contributing to responses to severe environmental conditions, which showed *OsNCED3* is the key regulator of ABA biosynthesis in rice. Therefore, *OsNCED3* is crucial to crop improvement. Moreover, future investigation of *OsNCED3* will include determining how *OsNCED* genes, together with *OsNCED3*, participate in other physiological functions that we could not observe in the *nced3* single mutant. Furthermore, other novel regulatory functions of *OsNCED3* could be studied by identifying its interacting proteins.

## Author contributions

YH and ML designed the experiments. YH performed the experiments and wrote the manuscript. YG, YL, and FZ generated the transgenic materials. ZW, FW, and HW provided assistance in the abiotic stress pre-screening experiment. DL, SL, and DM modified the manuscript. ML and LC analyzed the data and edited the manuscript. All authors read and approved the final manuscript draft.

### Conflict of interest statement

The authors declare that the research was conducted in the absence of any commercial or financial relationships that could be construed as a potential conflict of interest.
