# Supplementary material for: 9-cis-Epoxycarotenoid Dioxygenase 3 Regulates Plant Growth and Enhances Multi-Abiotic Stress Tolerance in Rice
Source: Front Plant Sci. 2018 Mar 6;9:162. doi: 10.3389/fpls.2018.00162 (PMC5845534; doi:10.3389/fpls.2018.00162)
Supplement: Supplementary file 1 [file Table1.DOCX]

**Table S1. CRISPR/Cas9-mediated target *OsNCED3* mutations in rice**

| No. of T_0_ plants | None-edited plants(%) | Edited plants(%) | | |
| --- | --- | --- | --- | --- |
|  |  | homozygous | bi-allelic | heterozygous |
| 35 | 3(8.6%) | 8(22.9%) | 2(3.7%) | 22(64.8%) |
