# Supplementary material for: 9-cis-Epoxycarotenoid Dioxygenase 3 Regulates Plant Growth and Enhances Multi-Abiotic Stress Tolerance in Rice
Source: Front Plant Sci. 2018 Mar 6;9:162. doi: 10.3389/fpls.2018.00162 (PMC5845534; doi:10.3389/fpls.2018.00162)
Supplement: Supplementary file 2 [file Table2.DOCX]

**Table S2. Analysis of potential off-target effects**

| Number of possible off target sites | Chrom | Position | Sequence | Mismatch | Region | No. of plants sequenced | No. of plants  with mutations |  |  |
| --- | --- | --- | --- | --- | --- | --- | --- | --- | --- |
| Off1 | 2 | 10124536 | GCCGCCCGCGCG**T**GCGC**A**GC**TGG** | 2 | intergenic | 35 | 0 |  |  |
| Off2 | 11 | 27576744 | GCCGCCCGCGCG**T**GCGC**A**GC**TGG** | 2 | CDS | 35 | 0 |  |  |
| Off3 | 9 | 12202128 | **T**CCGCCC**T**CGCGCGCGC**A**GC**CGG** | 3 | intergenic | 35 | 0 |  |  |
| Off4 | 6 | 4211319 | **A**CCGC**G**CGCGCGCGCGC**G**GC**CGG** | 3 | CDS | 35 | 0 |  |  |
| Off5 | 1 | 21115189 | **A**CCGCCCGC**C**CGCGCGCT**C**C**CGG** | 3 | CDS | 35 | 0 |  |  |
| Off6 | 1 | 28479538 | **C**CCGCCCGC**C**CGCGCGC**C**GC**CGG** | 3 | CDS | 35 | 0 |  |  |
| Off7 | 11 | 22805236 | GCCGCCCGCGCGCGCGC**GCTCGG** | 3 | intergenic | 35 | 0 |  |  |

The PAM motif (NGG) is green; mismatching bases are shown in red.
