# Supplementary material for: 9-cis-Epoxycarotenoid Dioxygenase 3 Regulates Plant Growth and Enhances Multi-Abiotic Stress Tolerance in Rice
Source: Front Plant Sci. 2018 Mar 6;9:162. doi: 10.3389/fpls.2018.00162 (PMC5845534; doi:10.3389/fpls.2018.00162)
Supplement: Supplementary file 3 [file Table3.DOCX]

**Table S3. Primers and oligos used in this study**

| Name | Primer sequence (5’-3’) |
| --- | --- |
| **Plasmid Constructions and Mutation Detection** | |
| U3-NCED3-F | GGCAGCCGCCCGCGCGCGCGCTGC |
| U3-NCED3-R | AAACGCAGCGCGCGCGCGGGCGGC |
| NCED3-J-F | ACGATCACGACGCCAGGATAT |
| NCED3-J-R | GCCTGCCGTTGAAGTAGATGAG |
| 1301-NCED3-F | AACTGCAGCACTTAGTCTACTTTGCACCTT |
| 1301-NCED3-R | CATGCCATGGAAGCATATCCTGGCGTCG |
| pHB-NCED3-F | AACTGCAGATGGCGACGATCACGACG |
| pHB-NCED3-R | GCTCTAGATCAGGCCTGGGTGGTGAG |
| pEZS-NCED3-F | CGGAATTCTGATGGCGACGATCACGACG |
| pEZS-NCED3-R | CGGGATCCACGGCCTGGGTGGTGAGCTC |
| Off1-F | CACTGTGCCCAATCTATACTTG |
| Off1-R | TTAACTTCTGGAGCATCG |
| Off2-F | TCGTCCTTCCTCCTGTT |
| Off2-R | TTTGATTAAGGGTGAGGTG |
| Off3-F | TCATCACTCGCCTACAATC |
| Off3-R | GCATCGCACCTTCTCA |
| Off4-F | CTCGTCCACTCATCATCG |
| Off4-R | CTTATCCAGATTTATCCTCCTA |
| Off5-F | AGGCCCGTCTGCGAGTCCAT |
| Off5-R | ACAGAGCAGCGACGGACAC |
| Off6-F | GTGTTGTCTGTGCGTGAG |
| Off6-R | TTGCCGTGGGAGTAGA |
| Off7-F | TTGTTGGTTGTAGGGTGTC |
| Off7-R | GCGGCGGAAGATGA |
| **qRT-PCR** |  |
| OsNCED3-F | CTCACATACAGCGGCAGCAC |
| OsNCED3-R | CGCTCGAGGACATTCGCCAC |
| Actin-F | CAATGTGCCAGCTATGTATGTCGCC |
| Actin-R | TTCCCGTTCAGCAGTGGTAGTGAAG |
| ABA8OX1-F | AAGCTGGCAAAACCAACATC |
| ABA8OX1-R | CCGTGCTAATACGGAATCCA |
| ABA8OX3-F | AGTACAGCCCATTCCCTGTG |
| ABA8OX3-R | ACGCCTAATCAAACCATTGC |
| ABI2-F | CGTTTGTGGACTGCTTCTCG |
| ABI2-R | CACGGTCTCGGGTGCTAC |
| PP2C68-F | CGCAGCTCCGACAACATCT |
| PP2C68-R | GCTGGGTGACACTCTCTCTACAAG |
| Rab21-F | CACACCACAGCAAGAGCTAAGTG |
| Rab21-R | TGGTGCTCCATCCTGCTTAAG |
| DREB2A-F | AGCACGTGTCAATTTTGCAG |
| DREB2A-R | CCATATTCTTCCGCTCCTGA |
| OsSGR-F | AGGGGTGGTACAACAAGCTG |
| OsSGR-R | GCTCCTTGCGGAAGATGTAG |
| OsNAP-F | CAAGAAGCCGAACGGTTC |
| OsNAP-R | GTTAGAGTGGAGCAGCAT |
| OsI85-F | GAGCAACGGCGTGGAGA |
| OsI85-R | GCGGCGGTAGAGGAGATG |
| OsNAC2-F | AAAAACAACCGCATTGGCAG |
| OsNAC2-R | AGTCCTCATCTCCTCTGTCTAATCC |
|  |  |
|  |  |
