# Supplementary material for: 9-cis-Epoxycarotenoid Dioxygenase 3 Regulates Plant Growth and Enhances Multi-Abiotic Stress Tolerance in Rice
Source: Front Plant Sci. 2018 Mar 6;9:162. doi: 10.3389/fpls.2018.00162 (PMC5845534; doi:10.3389/fpls.2018.00162)
Supplement: Supplementary file 4 [file Image1.PDF]

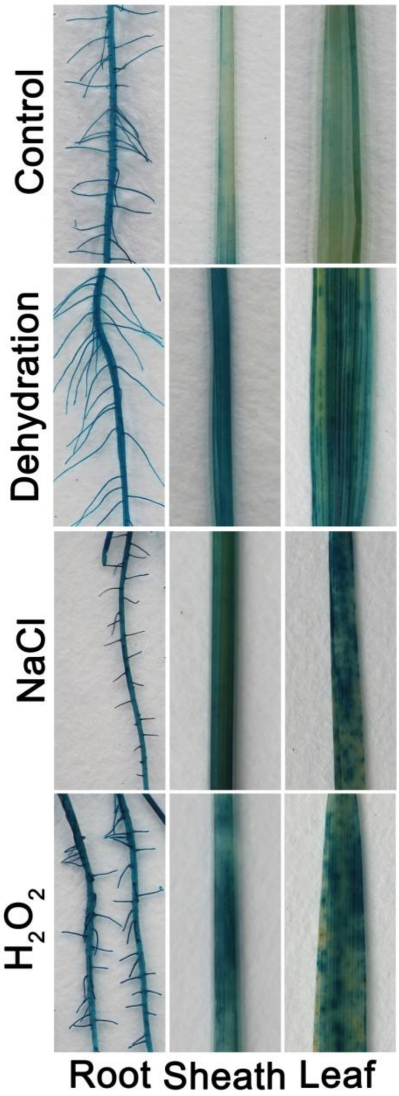

Figure S1 Histochemical staining of *OsNCED3*$_{Pro}$*::GUS* transgenic seedlings, including roots, sheaths and leaves under dehydration, NaCl and H_2O_2 stress, respectively.
