# Supplementary material for: 9-cis-Epoxycarotenoid Dioxygenase 3 Regulates Plant Growth and Enhances Multi-Abiotic Stress Tolerance in Rice
Source: Front Plant Sci. 2018 Mar 6;9:162. doi: 10.3389/fpls.2018.00162 (PMC5845534; doi:10.3389/fpls.2018.00162)
Supplement: Supplementary file 5 [file Image2.PDF]

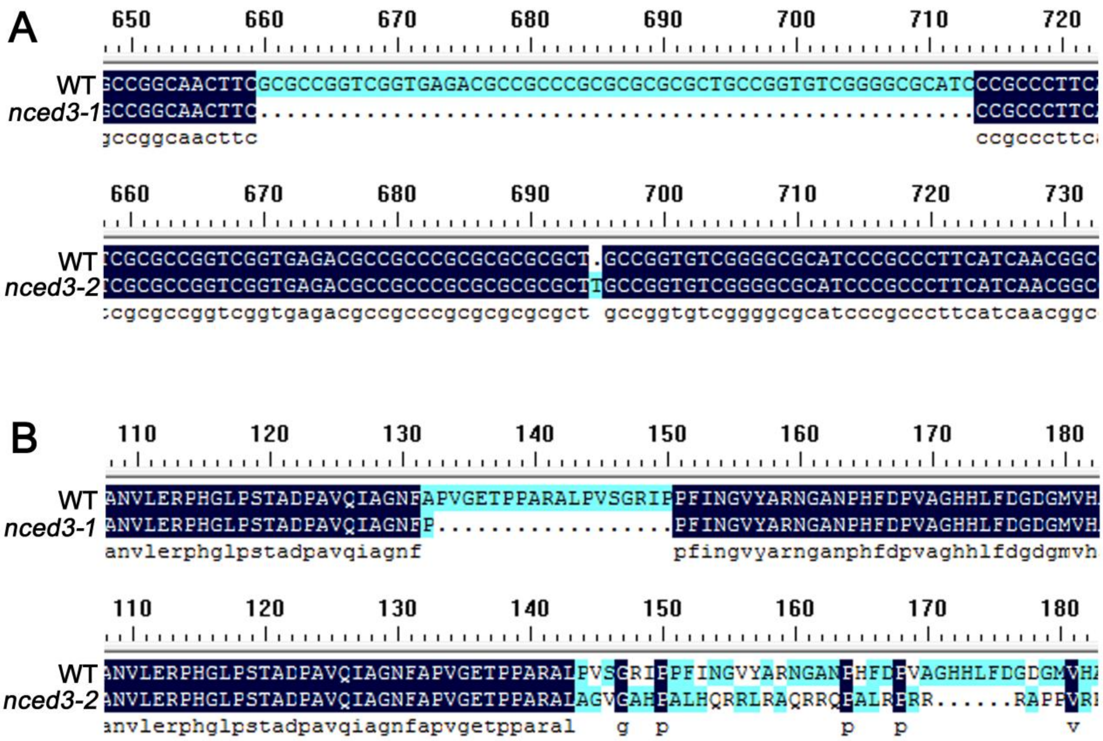

Figure S2 Sequence comparison of *nced3* mutants (*nced3-1* and *nced3-2*) and WT (Nipponbare) by DNAMAN software. (A) DNA sequence alignment of *nced3-1*, *2* and WT. (B) Amino acid sequence alignment of *nced3-1*, *2* and WT.
