# Supplementary material for: 9-cis-Epoxycarotenoid Dioxygenase 3 Regulates Plant Growth and Enhances Multi-Abiotic Stress Tolerance in Rice
Source: Front Plant Sci. 2018 Mar 6;9:162. doi: 10.3389/fpls.2018.00162 (PMC5845534; doi:10.3389/fpls.2018.00162)
Supplement: Supplementary file 6 [file Image3.PDF]

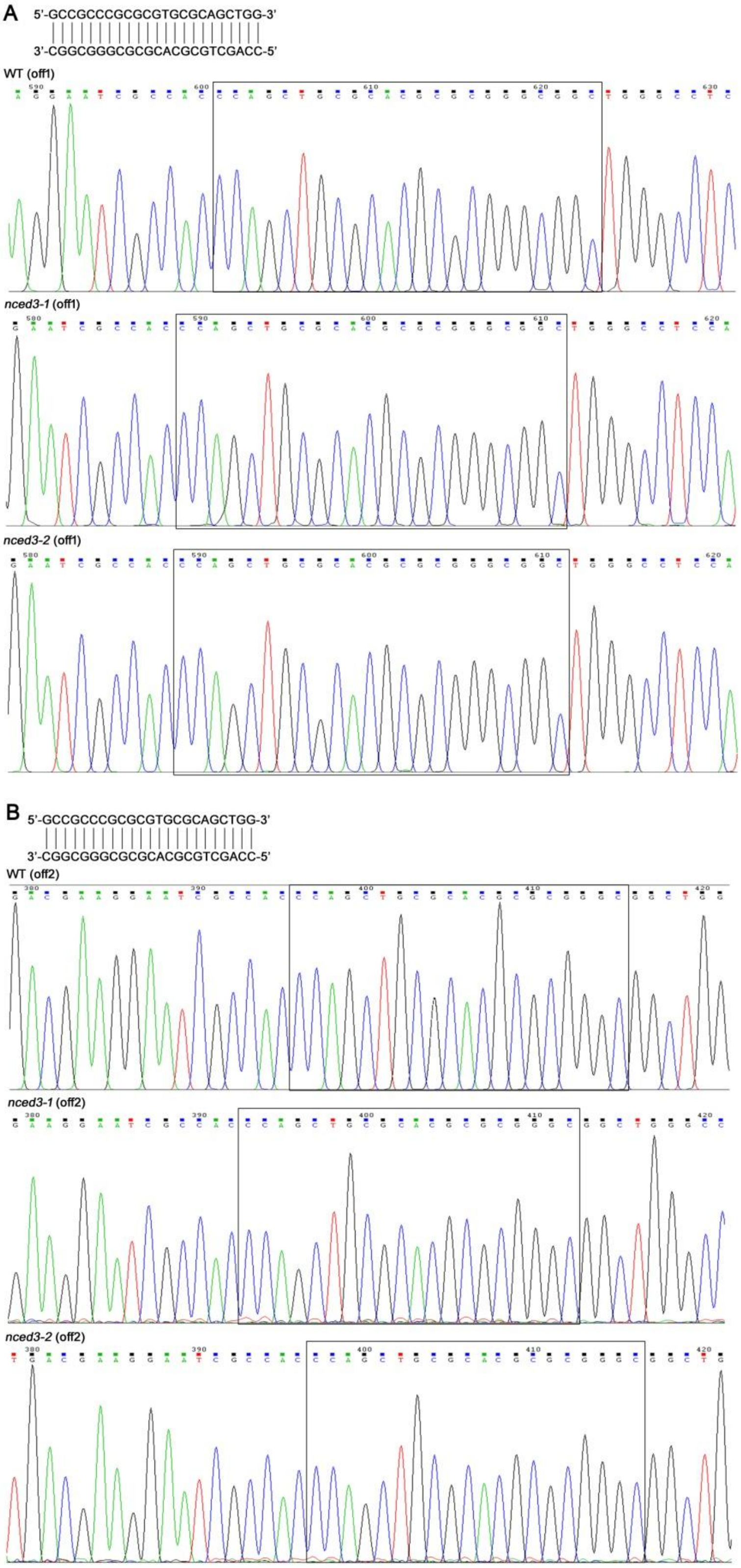
A
5'-GCCGCCCGCGCGTGCGCAGCTGG-3'
3'-CGGCGGGCGCGCACGCGTCGACC-5'
WT (off1)
nced3-1 (off1)
nced3-2 (off1)
B
5'-GCCGCCCGCGCGTGCGCAGCTGG-3'
3'-CGGCGGGCGCGCACGCGTCGACC-5'
WT (off2)
nced3-1 (off2)
nced3-2 (off2)

C

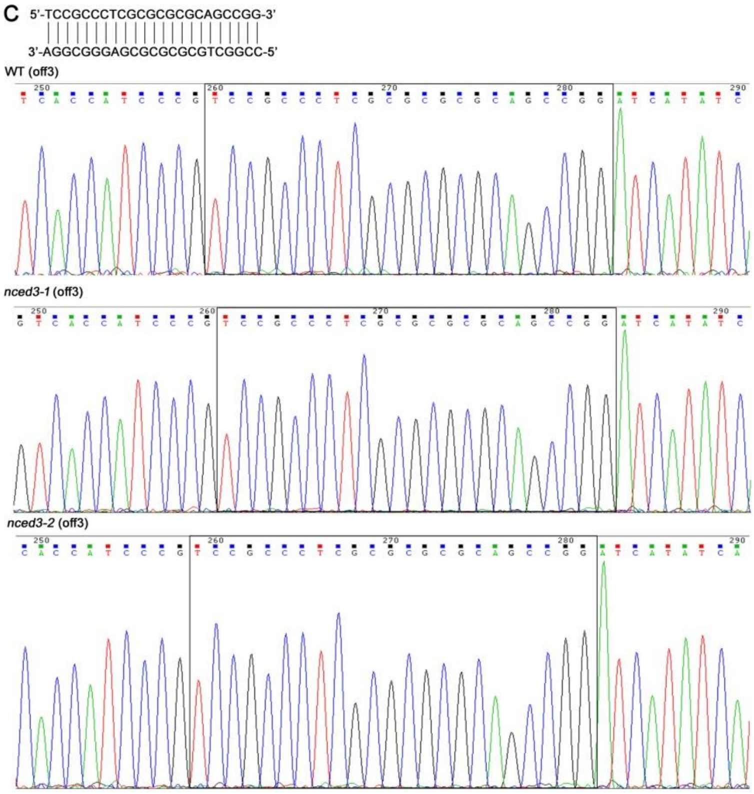

5'-TCCGCCCTCGCGCGCGCAGCCGG-3'
3'-AGGCGGGAGCGCGCGCGTCGGCC-5'
WT (off3)
nced3-1 (off3)
nced3-2 (off3)

D

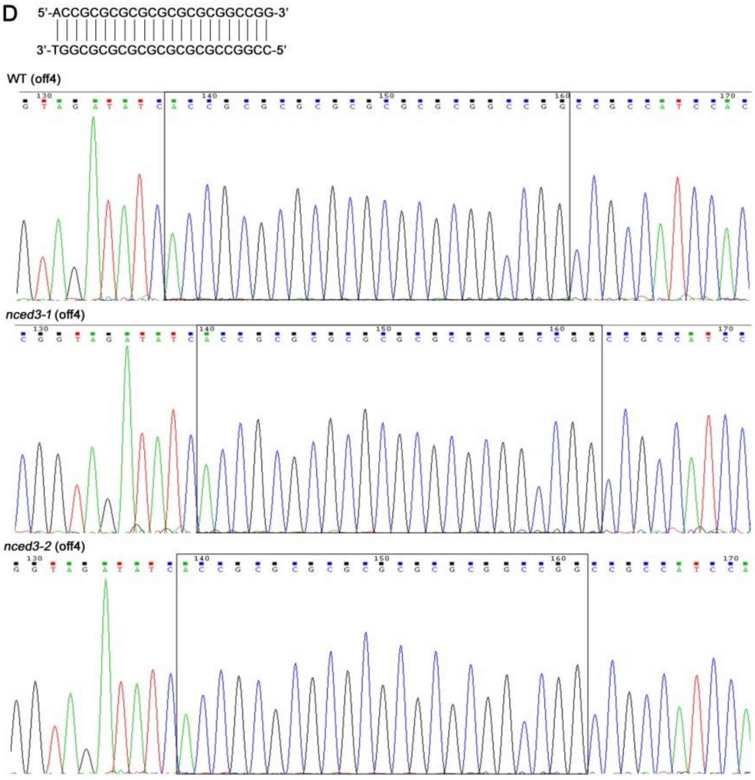

5'-ACCGCGCGCGCGCGCGCGGCCGG-3'
3'-TGGCGCGCGCGCGCGCGCCGGCC-5'
WT (off4)
nced3-1 (off4)
nced3-2 (off4)

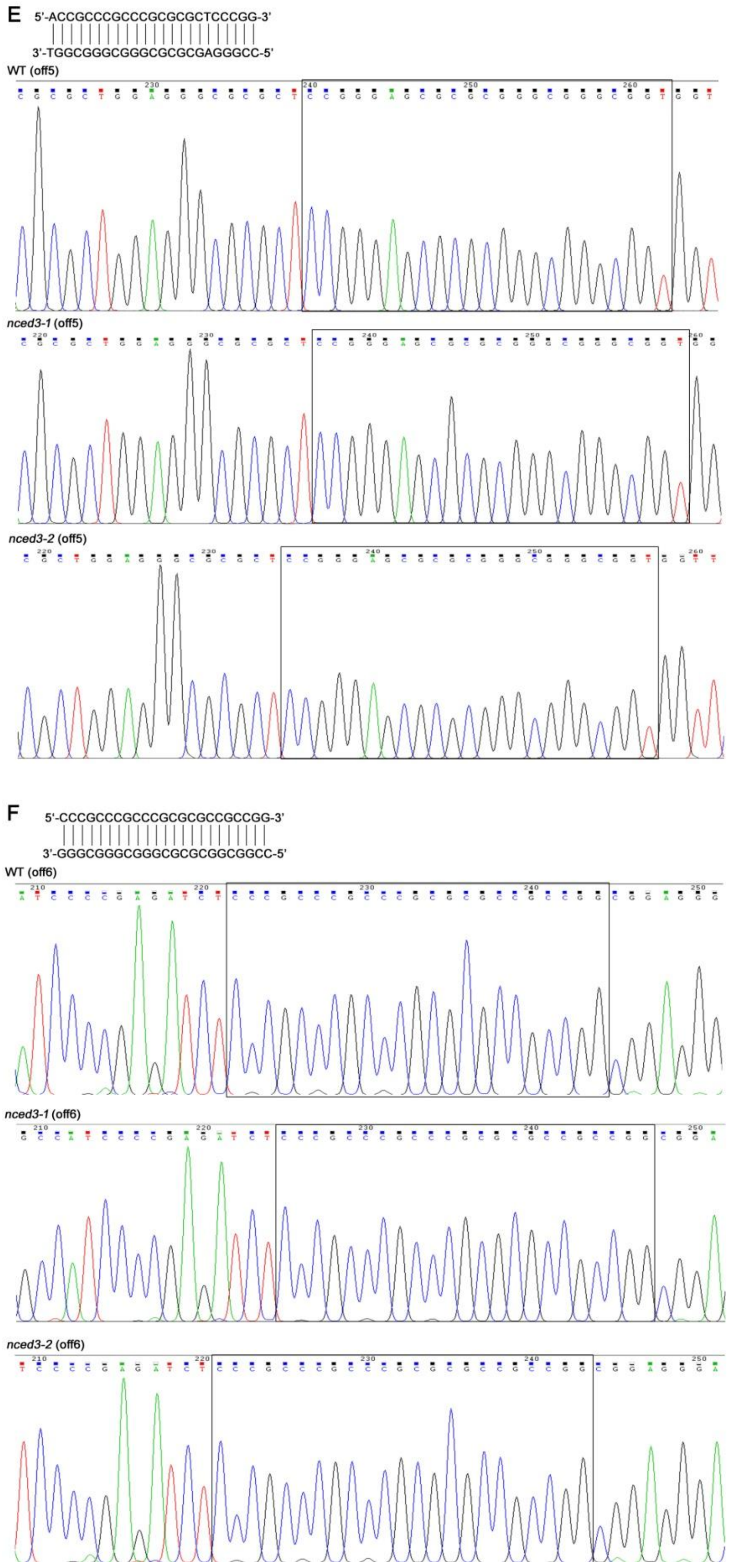
E
5'-ACCGCCCGCCCGCGCGCTCCCGG-3'
3'-TGGCGGGCGGGCGCGCGAGGGCC-5'
WT (off5)
nced3-1 (off5)
nced3-2 (off5)
F
5'-CCCGCCCGCCCGCGCGCCGCCGG-3'
3'-GGGCGGGCGGGCGCGCGGCGGCC-5'
WT (off6)
nced3-1 (off6)
nced3-2 (off6)

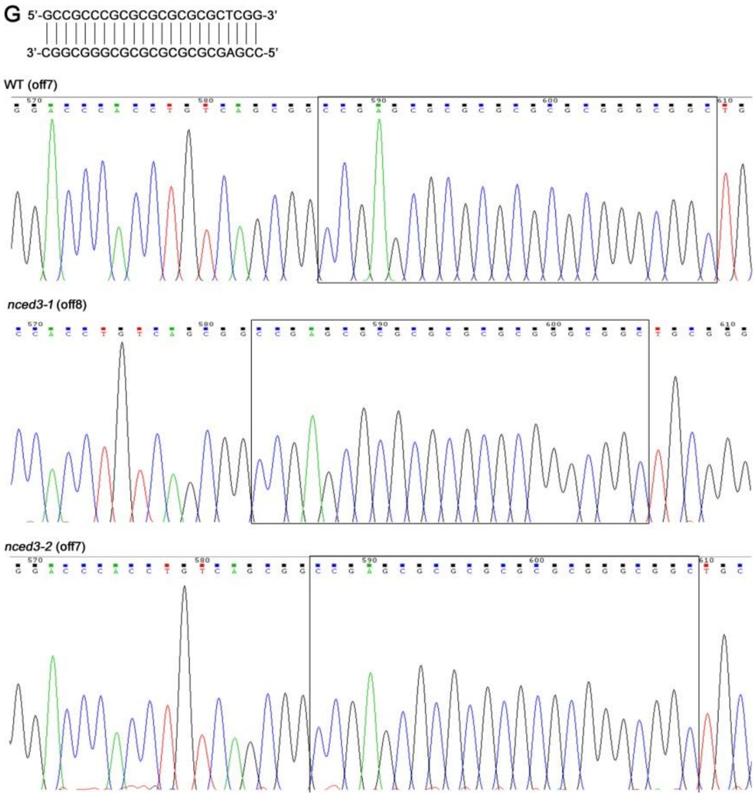

Figure S3. The sequencing chromatogram analyzed of seven potential off-target loci. A to G shows the sequencing chromatogram of WT, nced3-1 and nced3-2 of each locus. Rectangular region on the chromatogram high lights the potential off-terget locus.
