# Supplementary material for: 9-cis-Epoxycarotenoid Dioxygenase 3 Regulates Plant Growth and Enhances Multi-Abiotic Stress Tolerance in Rice
Source: Front Plant Sci. 2018 Mar 6;9:162. doi: 10.3389/fpls.2018.00162 (PMC5845534; doi:10.3389/fpls.2018.00162)
Supplement: Supplementary file 7 [file Image4.PDF]

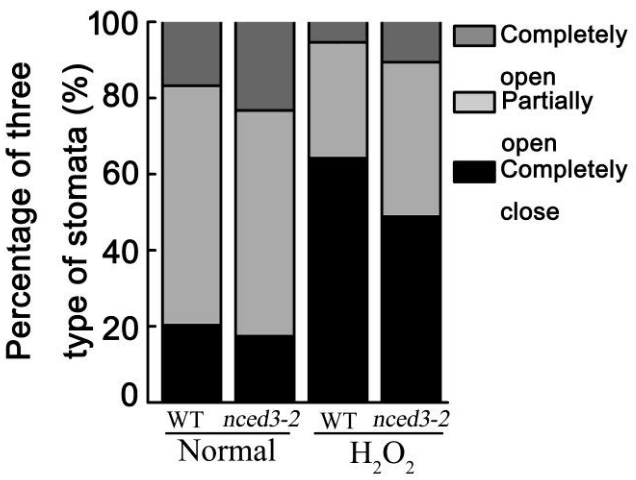

Figure S4 Stomata closure of *nced3* mutant under H_2O_2 stress. Scanning electron microscopy images of three levels of stomatal apertures, the percentage of three levels of stomatal apertures in *nced3-2* mutant and wild type plants were calculated under normal and H_2O_2 stress condition (n = 105 stomata for WT; n = 98 stomata for *nced3-2* mutant).
