# Supplementary material for: 9-cis-Epoxycarotenoid Dioxygenase 3 Regulates Plant Growth and Enhances Multi-Abiotic Stress Tolerance in Rice
Source: Front Plant Sci. 2018 Mar 6;9:162. doi: 10.3389/fpls.2018.00162 (PMC5845534; doi:10.3389/fpls.2018.00162)
Supplement: Supplementary file 8 [file Image5.PDF]

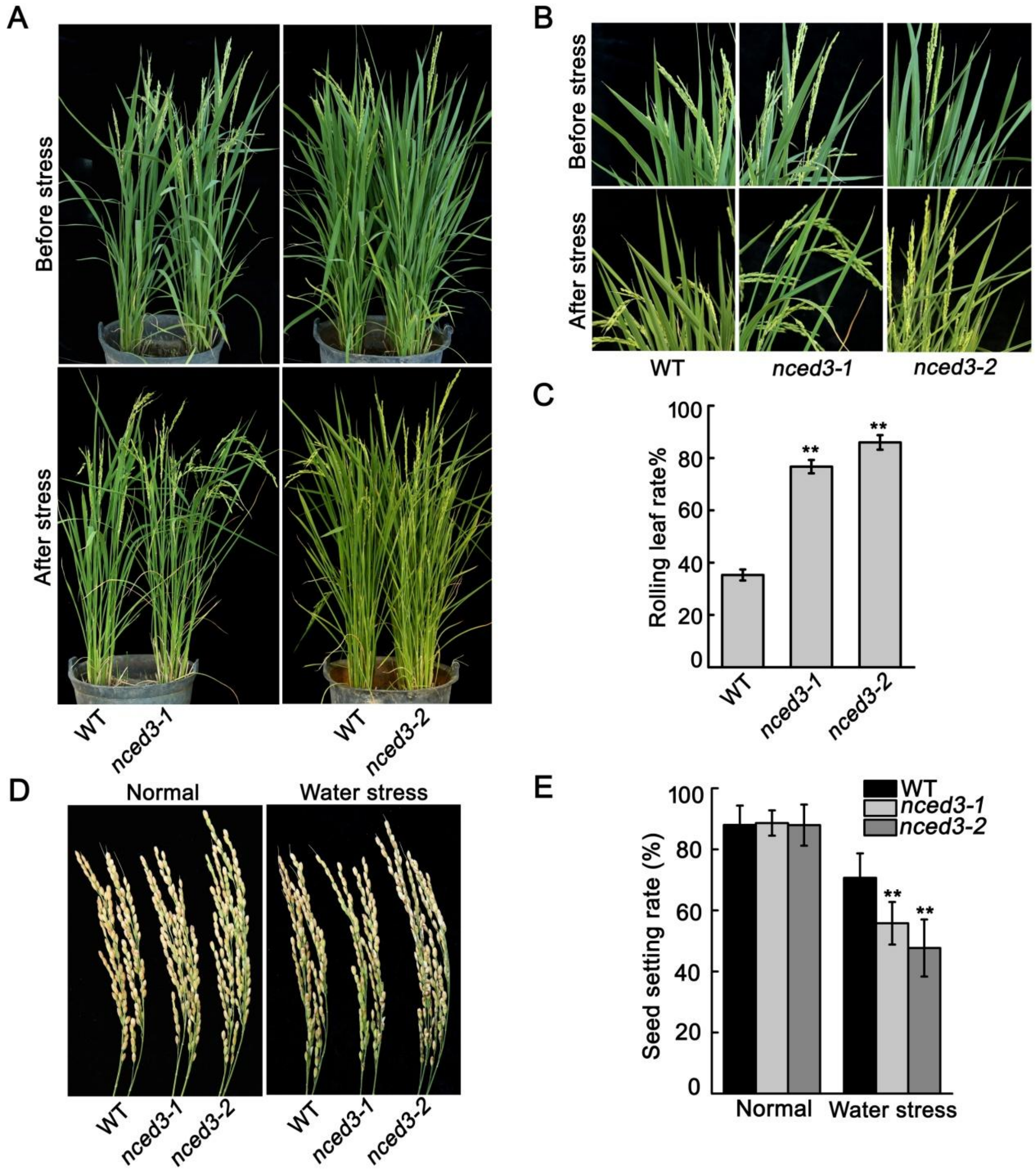

Figure S5 Drought tolerance testing of *nced3* mutant at the reproductive stage. (A, B) Phenotype of *nced3* mutant under water stress at the reproductive stage; (C) Rolling leaf rate after water stress. The number of rolling leaves as a proportion of the total leaves is shown. (D) Phenotype of *nced3* mutant seed setting after water stress; (E) Analysis of seed setting rate under normal condition and water stress. The similar result from three independent replicate.
