# Supplementary material for: 9-cis-Epoxycarotenoid Dioxygenase 3 Regulates Plant Growth and Enhances Multi-Abiotic Stress Tolerance in Rice
Source: Front Plant Sci. 2018 Mar 6;9:162. doi: 10.3389/fpls.2018.00162 (PMC5845534; doi:10.3389/fpls.2018.00162)
Supplement: Supplementary file 9 [file Image6.PDF]

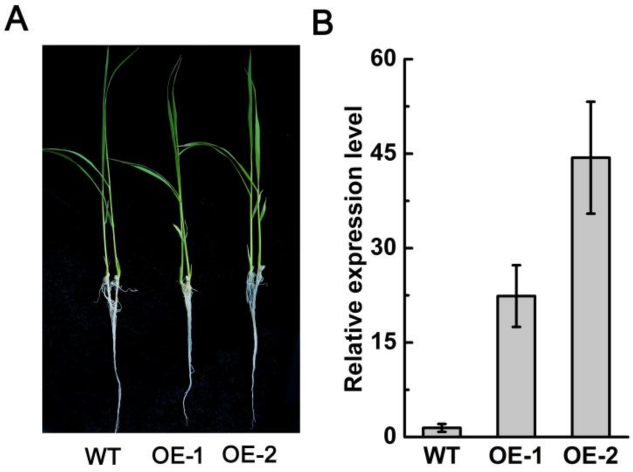

Figure S6 *OsNCED3*-overexpressing transgenic plants expression level analysis. (A) 3-leaf stage seedlings of *OsNCED3*-overexpressing transgenic plants. (B) Expression level analysis of *OsNCED3* gene in OE-1 and OE-2 transgenic plants. Data are mean ± SE for three replicates.
