# Supplementary material for: 9-cis-Epoxycarotenoid Dioxygenase 3 Regulates Plant Growth and Enhances Multi-Abiotic Stress Tolerance in Rice
Source: Front Plant Sci. 2018 Mar 6;9:162. doi: 10.3389/fpls.2018.00162 (PMC5845534; doi:10.3389/fpls.2018.00162)
Supplement: Supplementary file 10 [file Image7.PDF]

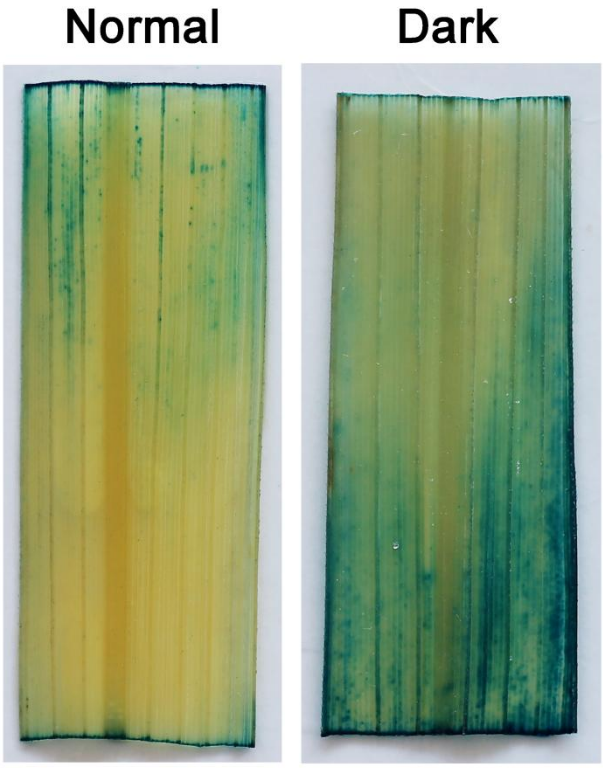

Figure S7 Histochemical staining of *OsNCED3*$_{Pro}$*::GUS* eight weeks old transgenic plants detached leaves after 3-day dark treatment.
